# A dsRNA-binding mutant reveals only a minor role of exonuclease activity in interferon antagonism by the arenavirus nucleoprotein

**DOI:** 10.1371/journal.ppat.1011049

**Published:** 2023-01-05

**Authors:** Patrick Bohn, Irke Waßmann, Lisa Wendt, Anne Leske, Thomas Hoenen, Birke A. Tews, Allison Groseth

**Affiliations:** 1 Institute of Molecular Virology and Cell Biology, Friedrich-Loeffler-Institut, Greifswald-Insel Riems, Germany; 2 Institute of Infectology, Friedrich-Loeffler-Institut, Greifswald–Insel Riems, Germany; University of Illinois at Chicago College of Medicine, UNITED STATES

## Abstract

The arenavirus nucleoprotein (NP) plays an important role in the virus’ ability to block interferon (IFN) production, and its exonuclease function appears to contribute to this activity. However, efforts to analyze this contribution are complicated by the functional overlap between the exonuclease active site and a neighboring region involved in IKKε-binding and subsequent inhibition of IRF3 activation, which also plays an important role in IFN production. To circumvent this issue, we mutated a residue located away from the active site that is involved in binding of the dsRNA substrate being targeted for exonuclease digestion, i.e. H426A. We found that expression of Tacaribe virus (TCRV) NP containing this RNA-binding H426A mutation was still able to efficiently block IFN-β promoter activity in response to Sendai virus infection, despite being strongly impaired in its exonuclease activity. This was in contrast to a conventional exonuclease active site mutant (E388A), which was impaired with respect to both exonuclease activity and IFN antagonism. Importantly, growth of a recombinant virus encoding the RNA-binding mutation (rTCRV-H426A) was similar to wild-type in IFN-deficient cells, unlike the active site mutant (rTCRV-E388A), which was already markedly impaired in these cells. Further, in IFN-competent cells, the TCRV-H426A RNA-binding mutant showed more robust growth and delayed IFN-β mRNA upregulation compared to the TCRV-E388A active site mutant. Taken together, this novel mutational approach, which allows us to now dissect the different contributions of the NP exonuclease activity and IKKε-binding/IRF3 inhibition to IFN antagonism, clearly suggests that conventional exonuclease mutants targeting the active site overestimate the contribution of the exonuclease function, and that rather other IFN antagonistic functions of NP play the dominant role in IFN-antagonism.

## Author summary

The ability to suppress interferon production plays an important role in the successful establishment of viral infection, and for arenaviruses, the exonuclease domain of the nucleoprotein is suggested to play an important role in this process. However, analyzing its contribution is challenging because mutations that disrupt the exonuclease active site also block antagonism of IRF3 activation, which is also important for interferon production. Here we report a mutation that instead targets binding of the exonuclease’s dsRNA substrate. As expected, this mutation strongly impaired exonuclease activity; however, unlike classical mutations targeting the exonuclease active site, it was still able to efficiently block interferon production. This suggests that data based on active site mutants overestimate the contribution of the NP exonuclease function to interferon antagonism. This advance in our understanding regarding the specific contributions of different NP functions to interferon antagonism has important implications for understanding arenavirus pathogenesis and the development of future treatment strategies.

## Introduction

The family *Arenaviridae* is comprised of viruses with a bi-segmented, ambisense single-stranded RNA (ssRNA) genome [1]. Those affecting mammals (i.e. genus *Mammarenavirus*) are divided into two groups, the New World (NW) and Old World (OW) arenaviruses, reflecting their genetic, antigenic and geographic relationships [2]. While both the NW and OW arenavirus groups include a number of important pathogens, among them Lassa virus (OW) and Junín virus (NW), many other arenaviruses exhibit little or no pathogenicity in humans [3]. In particular, the apathogenic Tacaribe virus (TCRV) is the prototype of the NW arenaviruses and is often used to study arenavirus biology under BSL 2 conditions and for comparative studies examining host cell responses to infection.

The type I interferon (IFN-I) system plays a critical role in defense against virus infections [4], and for arenaviruses this is particularly evident from the severe and lethal disease phenotypes observed when using IFN knock-out mice, despite the relative resistance of adult mice with an intact IFN system [5–8]. Indeed, arenavirus infections have been shown to induce IFN-I expression by several different mechanisms. This includes both binding of the viral glycoprotein to Toll-like receptor 2 [9], as well as the detection of viral RNA products by cytosolic RNA receptors. This leads to both RIG-I-dependent activation of IFN-I production, as well as PKR activation [10,11]. In particular for NW arenaviruses, the cytosolic accumulation of dsRNA has been directly shown to take place during infection [12]. Given the existence of these pathogen-associated molecular patterns (PAMPs) associated with arenavirus infection, it is then unsurprising that viral antagonists of IFN-I production have also been identified. The matrix protein (Z) has been shown to directly interact with RIG-I and MDA5 to block their downstream interaction with MAVS, and thereby inhibit the further signaling events needed for IFN-I induction [13,14]. Interestingly, however, it appears that only Z proteins of pathogenic arenaviruses, but not of apathogenic arenaviruses, are able to use this mechanism [14]. In contrast, the nucleoprotein (NP), in addition to its classical role in viral RNA synthesis, plays an important role in IFN antagonism for both pathogenic and apathogenic members of both the NW and OW arenavirus groups. Specifically, NP has been shown to interact via a DIEG(R) motif in its C-terminal domain with the kinase domain of IKKε, which then blocks TBK1-mediated phosphorylation of the transcription factor IRF3 [15–19]. In addition, Lymphocytic choriomeningitis virus (LCMV) NP has been suggested to interact directly with RIG-I and MDA5 to block IFN induction [20]. Further, structural studies of different arenavirus NPs have also recently revealed the existence of a conserved DEDDh family exonuclease (ExoN) domain within NP [21–24] that possesses functional 3’-5’ ExoN activity with a preference for dsRNA [22,23].

The DEDDh exonuclease domain structure includes four conserved acidic residues (i.e. aspartate and glutamate) and a nearby general base residue (i.e. histidine), and allows the conjugation of two divalent cations within the active site [25]. Despite their structural similarities, DEDDh exonucleases have varying degrees of specificity for single- or double-stranded templates composed of either RNA or DNA [26]. Based on structural data, the preference of the arenavirus NP ExoN for dsRNA has been suggested to be due to an unusual binding arrangement in which the RNA remains base-paired within the active site while various conserved residues make contact with either the substrate or non-substrate strands [23,27]. In particular, base-stacking interactions between a tyrosine residue (substituted by histidine in some arenavirus sequences, including TCRV) in NP and the non-substrate strand have been suggested to be important for dsRNA specificity [27].

A number of recent studies have indicated the relevance of the arenavirus ExoN activity in the control of IFN-I production in response to infection, and consequently this is currently suggested to be its primary function [15,19,28,29]. Specifically, NP appears to be able to facilitate the digestion of dsRNA species that would otherwise serve as PAMPs for cytosolic RNA sensors, such as RIG-I-like ligands and/or PKR [10,19,21,23,27,30]. However, one major caveat to the existing research on arenavirus ExoN function is that all studies to date have focused on mutation of the active site or its directly proximal residues [15,19,28,29]. Since the region forming the ExoN active site overlaps with the region responsible for IKKε interaction/IRF3 inhibition (reviewed in [31]), such mutations have the potential to influence both activities simultaneously. This makes efforts to evaluate the impact of the ExoN function of NP, independent of its already well-established role in IKKε-binding and IRF3 inhibition, challenging using such mutants. As such, new approaches for the creation of ExoN mutants are clearly needed. One potential solution to this issue might be the use of mutants targeting residues involved in substrate RNA-binding rather than the ExoN active site itself [23,27].

At the same time, while the IFN antagonistic functions of arenavirus NP have been shown to be broadly relevant for both NW and OW arenaviruses, regardless of pathogenicity, TCRV was originally noted as a possible exception [18]. This was initially attributed to atypical sequences at positions shown to be crucial for IFN antagonism in other arenaviruses, i.e. mutation of a GPPT loop directly following E388 of the DEDDh ExoN active site and overlapping the DIEG(R) motif involved in IKKε interaction/IRF3 inhibition [32]. Indeed, when the usual GPPT motif is present, TCRV NP shows both ExoN activity and robust IFN antagonism [23,33]. This has led to the suggestion that specific TCRV isolates may have lost their ability to antagonize IFN production, for instance as a result of repeated passaging for stock generation on cells that are IFN-deficient [33]. If this would indeed be the case, these TCRV isolates might then also represent an interesting model to study biological trade-offs associated with IFN antagonism, and to examine naturally-occurring virus isolates that are impaired in IFN antagonism.

To explore these possibilities towards generating additional resources for the investigation of the arenavirus NP ExoN function, we examined the principle feasibility of selecting for a TCRV variant containing the previously described mutations in the active site-proximal GPPT loop (GPPT>DLQL) both by analyzing the ability of these NP mutants to facilitate viral RNA synthesis in a minigenome system, as well as our ability to rescue the corresponding recombinant TCRVs. In addition, we also examined mutants in which the highly conserved G residue within the GPPT loop, which forms part of the region associated with IKKε-binding and IRF3 inhibition, was either selectively mutated or restored (i.e. G389A, G389P, DLQL>GLQL). For comparison, we analyzed mutants in which ExoN activity was abolished using a classical approach in which one of the key ExoN active site residues (E388A) was mutated. Finally, we generated a novel ExoN mutant based on mutation of H426A, which destabilizes binding of the non-substrate RNA strand without disrupting the ExoN active site or the region responsible for regulation of IKKε-binding/IRF3 inhibition. The E388A and H426A mutants were further compared with respect to virus growth, mutation stability, ExoN activity and their ability to inhibit IFN-β production either in a reporter assay or during virus infection.

## Results

### Construction of TCRV NP exonuclease mutants and calculation of their predicted stability

**Fig 1:**
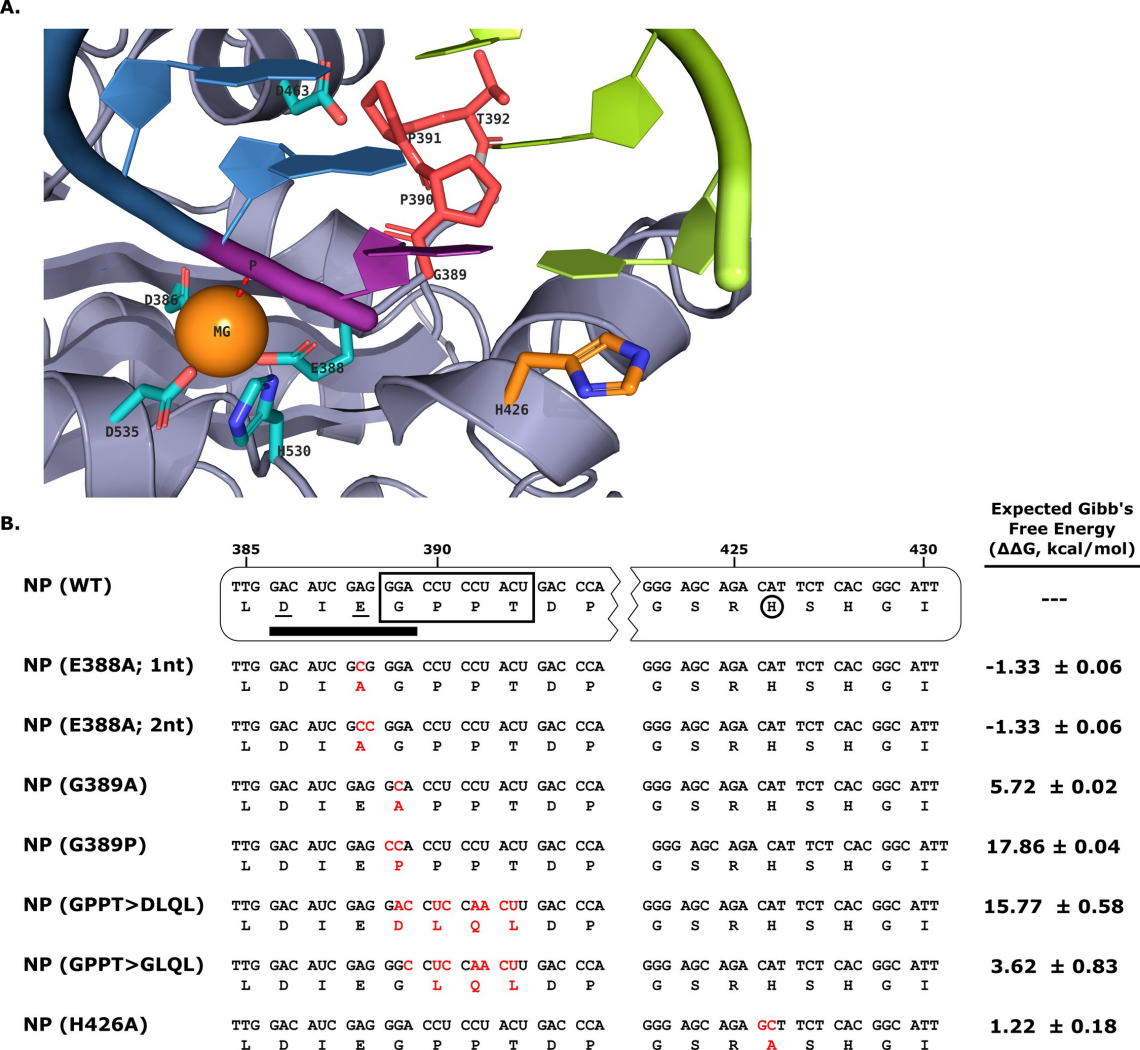
Construction of TCRV nucleoprotein NP exonuclease mutants. **(A) Structural model of the TCRV NP exonuclease active site.** A homology model of the TCRV NP C-terminal domain (PDB: 4GVE) based on the structure of the LASV NP CTD bound to dsRNA (PDB: 4VFU) is shown. Residues critical for exonuclease activity are shown in stick representation. Active site residues (DEDDh) are depicted in cyan and coordinate a divalent cation (shown here as Mg^2+^, orange). A GPPT sequence forming a flexible loop next to the active site is shown in red. The RNA substrate strand is colored in blue with the 3’-nucleotide to be cleaved in purple. The non-target strand of the substrate dsRNA (shown in green) stays associated with the target strand inside of the active site and interacts with the H426 residue via a base-stacking interaction. (**B) Sequence of TCRV NP mutants and assessment of their predicted impact on folding.** A sequence excerpt highlighting mutations (shown in red) in putative exonuclease mutants of TCRV NP constructed for this study. Residues that are part of the DEDDh exonuclease active site (i.e. D386 and E388) are shown underlined, the DIEG(R) motif associated with IKKε binding and inhibition of IRF3 activation is indicated by a black bar, and the H426 residue associated with non-template strand RNA-binding is circled. A flexible loop sequence located next to the exonuclease active site that is reported to be mutated from GPPT to DLQL by a pair of frameshift mutations in some TCRV isolates is shown boxed. The expected change in Gibb’s free energy (ΔΔG) is shown for each mutant relative to wild-type.

In order to generate TCRV NP mutants with impaired ExoN activity, mutations were introduced in one of three positions: 1) at the metal ion-binding site E388 that forms part of the NP DEDDh ExoN active site (i.e. E388A); 2) at the highly conserved residue G389 in the active site-proximal loop that has been previously reported to impact interaction with IKKε (i.e. G389A, G389P); 3) at position H426, which has been shown to participate in binding of the substrate dsRNA, through its non-substrate strand, outside the ExoN active site (i.e. H426A) (Fig 1A). In addition, TCRV NP mutants were constructed containing the previously reported DLQL sequence at amino acids positions 389–392 (i.e. GPPT>DLQL) or where the conserved G residue in this sequence was restored (GPPT>GLQL).

Free energy predictions for these mutants using the FoldX BuildModel prediction tool suggested only minimal changes in stability resulting from either the E388A or H426A mutations (Fig 1B). However, mutations to the active site-proximal loop (i.e. G389A, G389P, GPPT>DLQL or GPPT>GLQL) resulted in a marked increase in Gibb’s free energy values, suggesting reduced stability of these mutants compared to wild-type (Fig 1B). Notably, both the exchange of the conserved G389 residue and changes in the proline content of this loop region appear to be structurally unfavorable.

### Expression of TCRV NP exonuclease mutants and their activity in a minigenome assay

**Fig 2:**
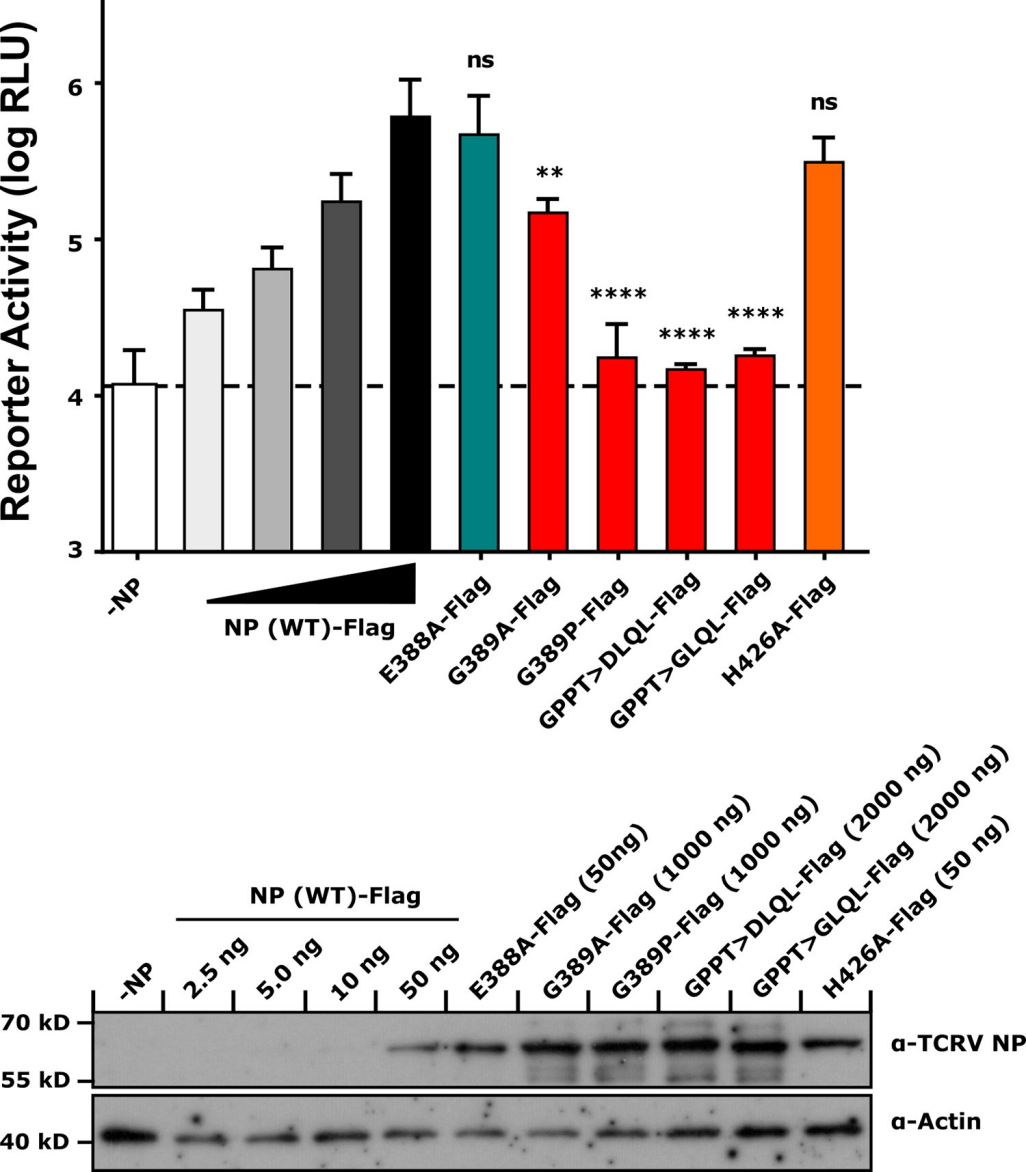
Activity of TCRV NP exonuclease mutants in a minigenome assay. BSR-T7/5 cells were transfected with plasmids expressing the S segment-based TCRV minigenome (125ng), T7 RNA polymerase (125ng), TCRV L (500ng) and Firefly luciferase (50ng, for normalization). Samples were additionally transfected with either Flag-tagged TCRV NP wild-type (WT) or a Flag-tagged TCRV NP mutant, as indicated. The amount of transfected NP (WT) plasmid was increased up to 50 ng, which was determined to result in maximal levels of reporter activity (S1 Fig), while amounts of plasmids encoding NP mutants was variable according to their expression. Cells were lysed and luciferase activity measured 48 h post-transfection. Reporter activity is shown and was calculated by normalizing Nanoluciferase activity to Firefly activity (top panel). Means and standard deviations are shown from four independent replicates. Statistical analysis was performed using a one-way ANOVA with Dunnett’s post-hoc test for differences compared to TCRV NP (WT)-Flag (50 ng) (ns–not significant, ** p≤0.01, **** p≤0.0001). Cell lysates were further examined for expression of TCRV NP or its mutants by Western blot using a polyclonal guinea pig anti-TCRV NP antibody, with detection of actin serving as a loading control.

To assess the functionality of these various TCRV NP mutants, their activity was tested in a TCRV minigenome assay, which models viral RNA synthesis. In establishing this assay, we observed that assay performance was very similar regardless of whether an S-segment or L-segment based minigenome was used (S1 Fig). Further, the addition of a Flag-tag to either the N- or C-terminus of NP did not significantly impact viral RNA synthesis, and thus would also not be expected to affect the activity of our mutants in subsequent experiments where the tag serves to allow standardization of protein expression. Interestingly, addition of an N-terminal combined Flag/HA-tag modestly decreased reporter activity, although it does not appear to affect protein expression (S1 Fig, panel B). Importantly, we could show that reporter activity increases with the transfection of increasing amounts of plasmid encoding TCRV NP, but only up to 12.5ng of transfected plasmid (S1 Fig, panel C). Above this amount activity remains at a maximum independent of further increases in NP expression. Based on these data we proceeded to use an S-segment based minigenome for analysis of our respective mutants with increasing amounts of NP (WT)-expressing plasmid used, up to 50ng. Interestingly, while protein expression levels based on transfection of an equivalent amount of plasmid was comparable for both the classical ExoN active site mutant (E388A) and the substrate RNA-binding mutant (H426A) (Fig 2, bottom panel and S2 Fig, panel A), for the other mutants affecting the active site-proximal loop, expression was markedly reduced. Indeed, in order to obtain protein expression levels that were at least equal to those of the NP (WT)-Flag we had to transfect 20-40x as much plasmid (Fig 2, bottom panel and S2 Fig, panel A). These expression data appear to correlate with the predicted changes in protein stability, based on the respective free energy values calculated for these mutants (c.f. Fig 1B). Further, while both the active site mutant (E388A) and the substrate RNA-binding mutant (H426A) showed levels of viral RNA synthesis comparable to wild-type, mutations in the active site-proximal loop resulted in NP mutants with markedly decreased levels of minigenome reporter activity (Fig 2, top panel). Specifically, while the G389A mutation was to some extent tolerated, albeit with a significant reduction in activity, other mutations affecting the proline-content of this loop (i.e. G389P, GPPT>DLQL, GPPT>GLQL) showed levels of reporter activity similar to samples without NP (Fig 2, top panel).

### In vitro exoribonuclease activity of TCRV NP and its mutants

Next, to compare the ExoN activity of different TCRV NP mutants we first established a non-radioactive ExoN assay for arenavirus NP. This approach was based on similar assays that have been developed for the study of pestivirus exonucleases [34] but was modified to accommodate the cation and substrate preferences of the arenavirus NP. Once these adjustments were made, ExoN activity could be clearly observed for TCRV NP, as well as JUNV NP and LASV NP (S3 Fig, panel A). Significant increases in free nucleotide release were observed in most cases starting after 4h of incubation, and continued to increase with prolonged reaction times out to 24h (S3 Fig, panel A). Importantly, TCRV NP remained functional following addition of a C-terminal Flag-tag, and indeed there was no statistically significant difference between free-nucleotide release between NP (WT) with and without a Flag-tag at any time point tested (S3 Fig, panel B).

**Fig 3:**
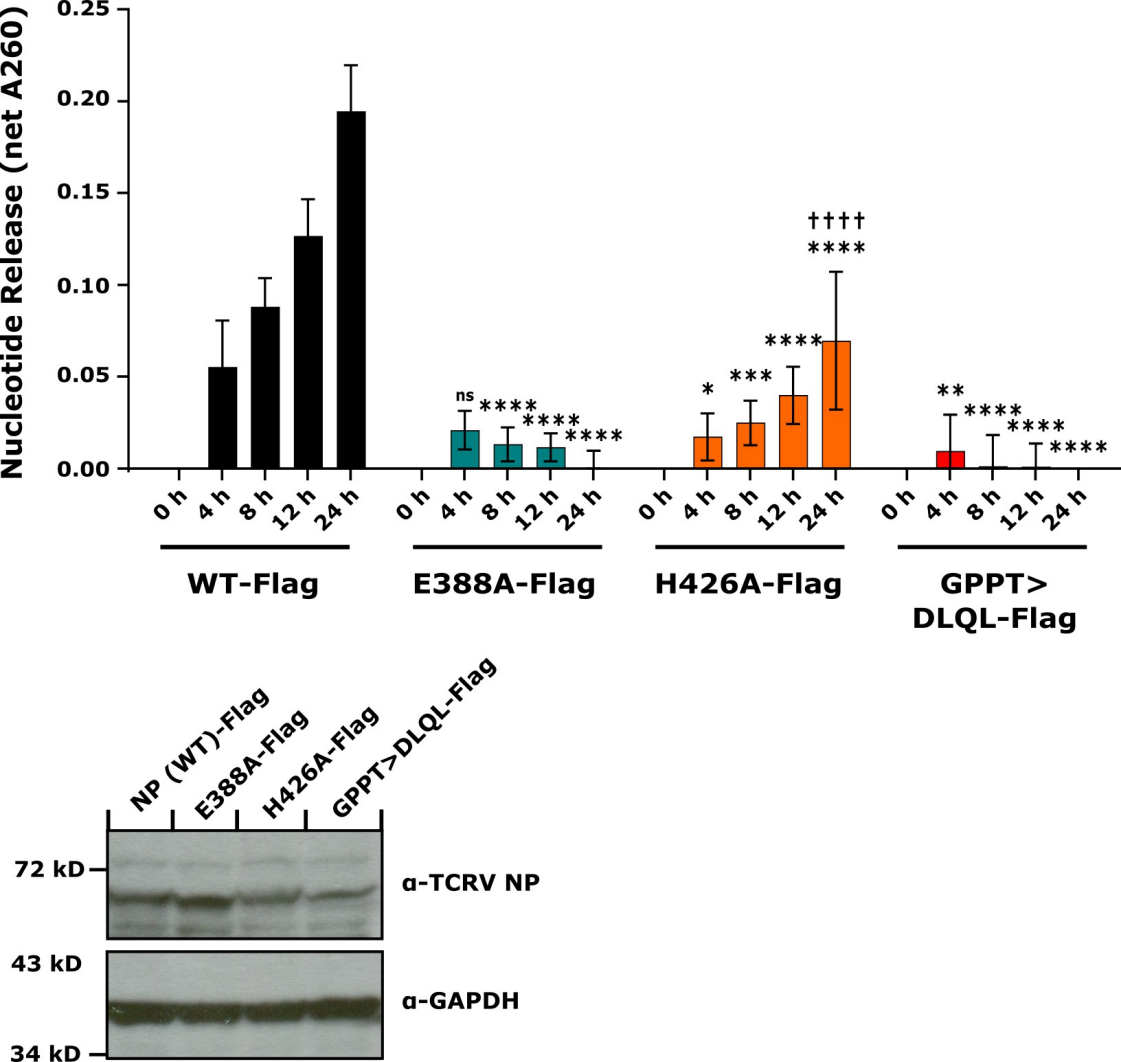
*In vitro* exoribonuclease activity of TCRV NP and its mutants. HEK 293T cells were transfected with plasmids encoding either Flag-tagged TCRV NP or its mutants, as indicated. After 3 d cell lysates were harvested and incubated with the dsRNA substrate poly(I:C). Release of free nucleotides (A260) was then measured at the indicated time points. Background values for individual samples (at 0 h) and for a negative control sample (i.e. without NP) were subtracted from values to calculate the net A260 value for each sample (upper panel). Data are shown as means and standard deviations of three independent replicates. Statistical analysis was performed using two-way ANOVA with Tukey’s post-hoc test for differences compared to TCRV NP (WT)-Flag (indicated with stars; ns—not significant, * p≤0.05, ** p≤0.01, *** p≤0.001, **** p≤0.0001) or between TCRV NP(E388A)-Flag, and TCRV NP(H426A)-Flag, indicated with daggers (†††† p≤0.0001, all other pairings were not significant). Cell lysates were further analyzed by Western blot to detect the amount of NP expressed in the respective samples using a polyclonal guinea pig anti-TCRV NP antibody and with GAPDH stained as a loading control (lower panel).

Based on these assay conditions, we next examined the ExoN activity of our mutants based either on disruption of the active site (E388A) or substrate RNA-binding (H426A). As a negative control we also examined the ExoN activity of the GPPT>DLQL mutant, which has been previously reported to be impaired in its ability to inhibit the IFN pathway and for which our data suggest it may be misfolded, consistent with its complete lack of activity in RNA synthesis and impaired expression (c.f. Fig 2). As expected, we found that all three mutants had a strongly reduced ExoN activity compared to TCRV NP (WT), with the GPPT>DLQL mutant showing a 97% loss of activity, the E388A mutant showing an 85% loss of activity, and the H426A mutant showing a 68% loss of activity. Importantly, while the H426A mutant clearly showed a greater degree of residual activity that the other mutants, this was still at a level that was greatly reduced compared to wild-type (Fig 3, top panel). Indeed, only after a reaction time of 24h could levels of free nucleotide release be detected with H426A-containing cell lysates that significantly exceeded those containing the E388A mutant (Fig 3, top panel). These differences were despite comparable levels of protein expression in the cell lysates tested (Fig 3, bottom panel and S2 Fig, panel B).

As a proof-of-principle looking at extending the utility of our non-radioactive ExoN assay (e.g. for high-throughput applications), we further adapted this method to a completely 96-well plate-based format. In this format the assay retained robust differences between positive (i.e. TCRV NP(WT)) and negative (i.e. TCRV NP(E388A)) samples, and generated an initial Z’-factor of 0.24 (S4 Fig, panel A). Further, using this approach we could show that nucleotide release by TCRV NP in this assay was strongly blocked by the known nuclease inhibitor Aurintricarboxylic acid (ATA) at concentrations of 10uM and above (S4 Fig, panel B), which is broadly consistent with previous reports for the inhibition of other arenavirus NPs [35,36]. Importantly, in addition to supporting the potential utility of this assay for drug-screening applications, it also confirms that nucleotide release in this assay is a specific result of ExoN activity by TCRV NP.

### Inhibition of IFN-β reporter plasmid activation by TCRV NP and its mutants

**Fig 4:**
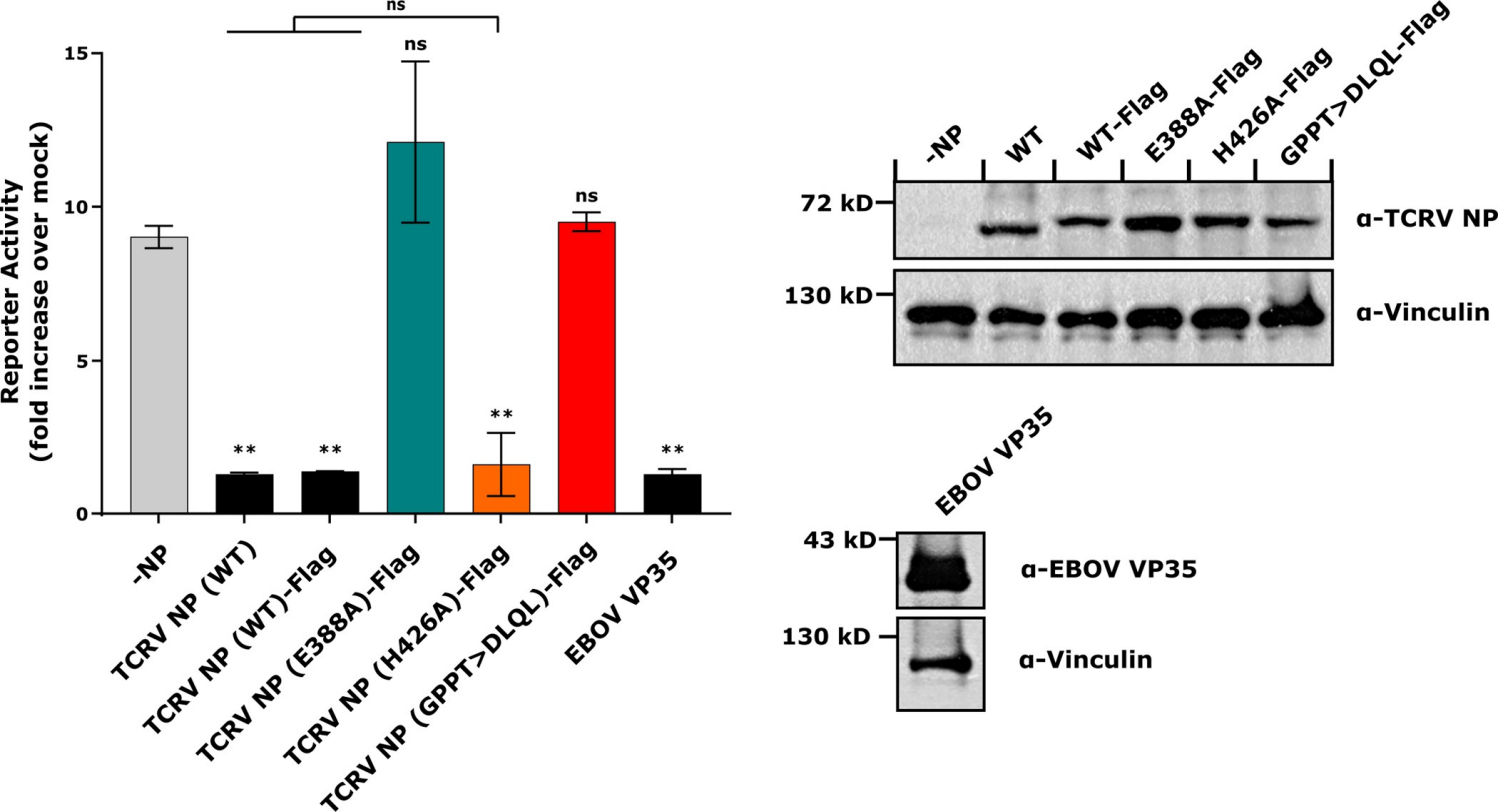
Inhibition of IFN-β reporter plasmid activation by TCRV NP and its mutants. HEK 293T cells were pre-transfected with a plasmid encoding firefly luciferase under the control of a human IFN-β promotor, as well as plasmids for either untagged TCRV NP, Flag-tagged TCRV NP, or its mutants, or EBOV VP35 (as a positive control), as indicated. After 24 h cells were either infected with Sendai virus at MOI = 200, or mock infected. At 24 h post-infection cells were lysed and luciferase activity was measured. Fold induction was calculated based on replicate samples that were either infected or left uninfected for each sample (left panel). Data are shown as means and standard deviations of two independent replicates. Statistical analysis was performed using one-way ANOVA with Dunnett’s post-hoc test for differences compared to the control without TCRV NP (-NP) (ns = not significant, ** p≤0.01) or Tukey’s post-hoc test for differences compared to TCRV NP (WT) or TCRV NP (WT)-Flag (ns—not significant). Cell lysates were further analyzed by Western blot to detect the amounts of NP expressed in the respective samples using a polyclonal guinea pig anti-TCRV NP or polyclonal rabbit anti-EBOV VP35 antibody, and with vinculin stained as a loading control (right panel).

Given that there is an overlap between the regions associated with formation of the ExoN active site (i.e. E388A), the active site-proximal loop (i.e. GPPT) and the regions associated with inhibition of IFN production at the level of inhibition of IRF3 activation (i.e. DIEG(R)) [15–19] (Fig 1), we further wanted to compare the ability of these mechanistically different ExoN mutants to inhibit signaling through the IFN production pathway. To do this, we tested each of them in an IFN-β reporter assay using Sendai virus (SeV) infection as a source of dsRNA to trigger signaling through the activation of RIG-I-like ligands. As expected, we could show robust inhibition of reporter expression following transfection of TCRV-NP (WT) either with or without a Flag-tag as well as another well described antagonist of IFN production, Ebola virus VP35 (Fig 4, left panel). We saw complete loss of this inhibitory activity with both the E388A active site mutant and the GPPT>DLQL mutant, as previously reported (Fig 4, left panel) [15,19,23,33]. In contrast, we found that despite being strongly inhibited in its ExoN activity, the H426A mutant retained the ability to inhibit the IFN production pathway to nearly the same extent as NP(WT) (i.e. 88% of WT inhibition; Fig 4, left panel). This difference in IFN antagonism phenotype was observed despite equivalent expression of all NP mutants (Fig 4, right panel and S2 Fig, panel C). Taken together, these data strongly suggest that the ExoN activity of TCRV NP plays only a minor role in contributing to its IFN antagonism.

### Interaction of TCRV NP and its mutants with IKKε

**Fig 5:**
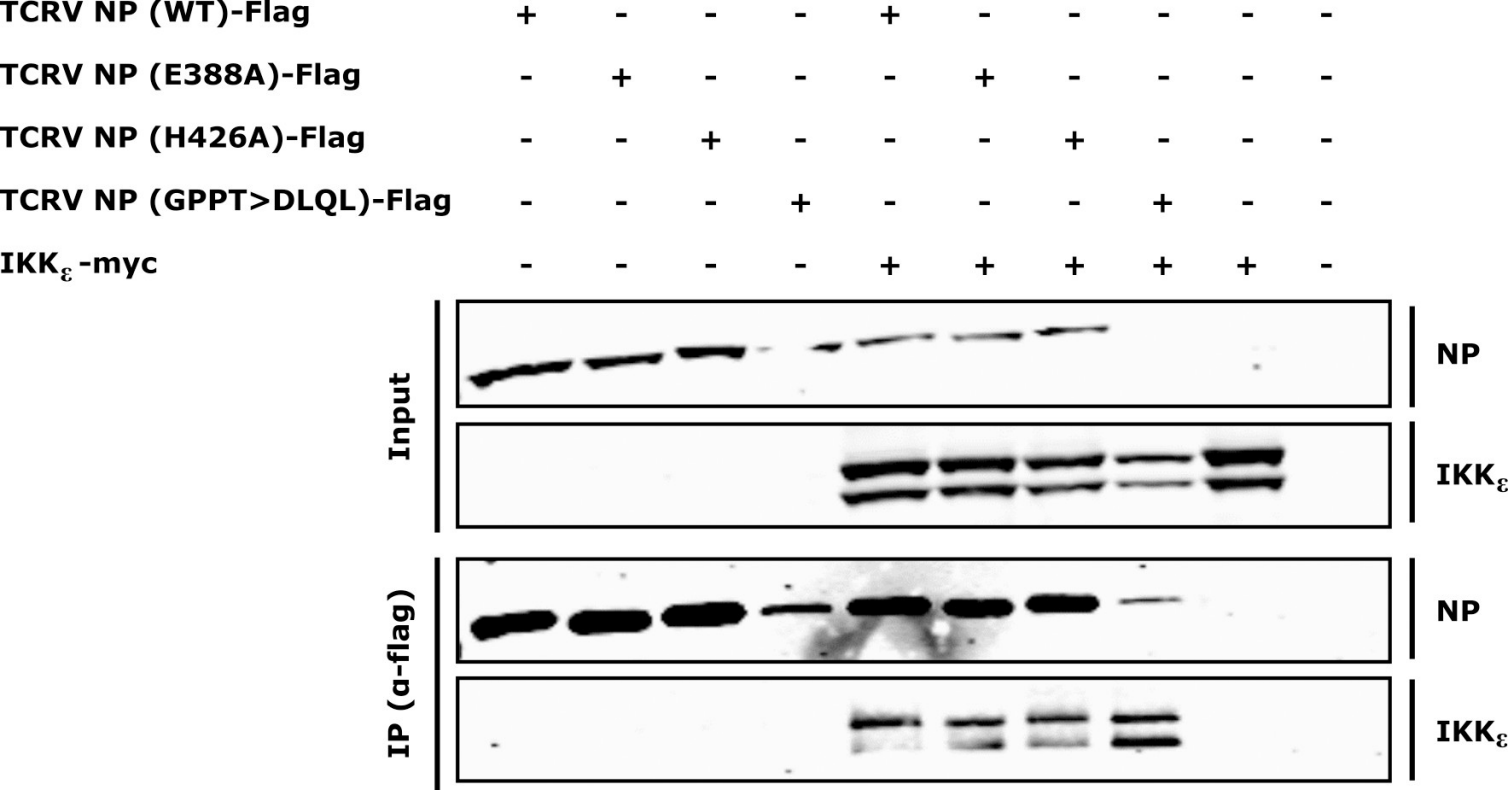
Co-immunoprecipitation of TCRV NP and exonuclease mutants with IKKε. HEK 293T cells were transfected with plasmids encoding Flag-tagged TCRV NP or its mutants, as well as myc-tagged IKKε, as indicated. Two days post-transfection cells were lysed and TCRV NP was precipitated with an anti-Flag antibody and input as well as precipitates were analyzed by Western blot. NP and IKKε were detected with antibodies specific for the Flag- or myc-tag, respectively. A representative result from 3 independent experiments is shown.

As noted, one possible explanation for the differences in IFN antagonism between the active site mutant (E388A) and the RNA substrate-binding mutant (H426A) is that amino acids involved in formation of the active site (i.e. D386 and E388) overlap with a region involved in inhibition of IRF3 activation (i.e. DIEG(R)). Indeed, for both Pichinde virus (PICV) and LCMV NP, mutation of active site residues has been shown to also impact interaction of NP with the IRF3 regulatory subunit IKKε [16,19]. However, when we examined interaction of NP with IKKε by co-immunoprecipitation, we did not see differences in the extent of interaction between TCRV NP(WT) and the E388A or H426A mutants (Fig 5). Again here, protein expression between these mutants was comparable (S2 Fig, panel D). Further, even the GPPT>DLQL mutant, which our data indicate may show major structural changes, while poorly expressed (S2 Fig, panel D), nonetheless continued to pull-down as much or more IKKε than other mutants, suggesting enhanced interaction with IKKε (Fig 5). To exclude a non-specific interaction between TCRV NP and the myc-tag used to detect IKKε, we confirmed that an unrelated myc-tagged protein (Ebola virus VP30) cannot be precipitated by TCRV NP or any of its mutants (S5 Fig). These data then clearly suggest that, for TCRV NP, changes in IKKε interaction are not responsible for the observed differences in the ability of the various ExoN mutants to inhibit IFN-β signaling in a reporter assay.

### Characterization of recombinant Tacaribe viruses (TCRVs) encoding exonuclease mutants of NP for growth & IFN-β mRNA induction

Finally, in order to be able to further characterize the biological consequences of impaired ExoN activity achieved by these different mutations in a viral context, a full-length clone system was used to rescue the corresponding recombinant viruses. Consistent with the minigenome assay data, which showed a complete loss of activity in viral RNA synthesis, a GPPT>DLQL mutant could not be rescued. In contrast, for TCRV NP(E388A) and (H426A), both of which showed levels of RNA synthesis comparable to NP(WT), recombinant viruses containing these mutants could be readily rescued. However, when the E388A mutation was introduced using only a single nucleotide change (GAG>GCG) we observed that the virus rapidly reverted to wild-type during further passaging/propagation (S6 Fig), despite these procedures being performed in IFN-I-deficient Vero76 cells. In contrast, a mutation generated based on the exchange of 2 nucleotides (GAG>GCC) remained stable over 5 passages in Vero76 cells (S6 Fig), and consequently this mutant was used for further characterization. No issues with reversion of the H426A mutant, which was also based on changes in 2 nucleotides (CAT>GCT), were noted.

**Fig 6:**
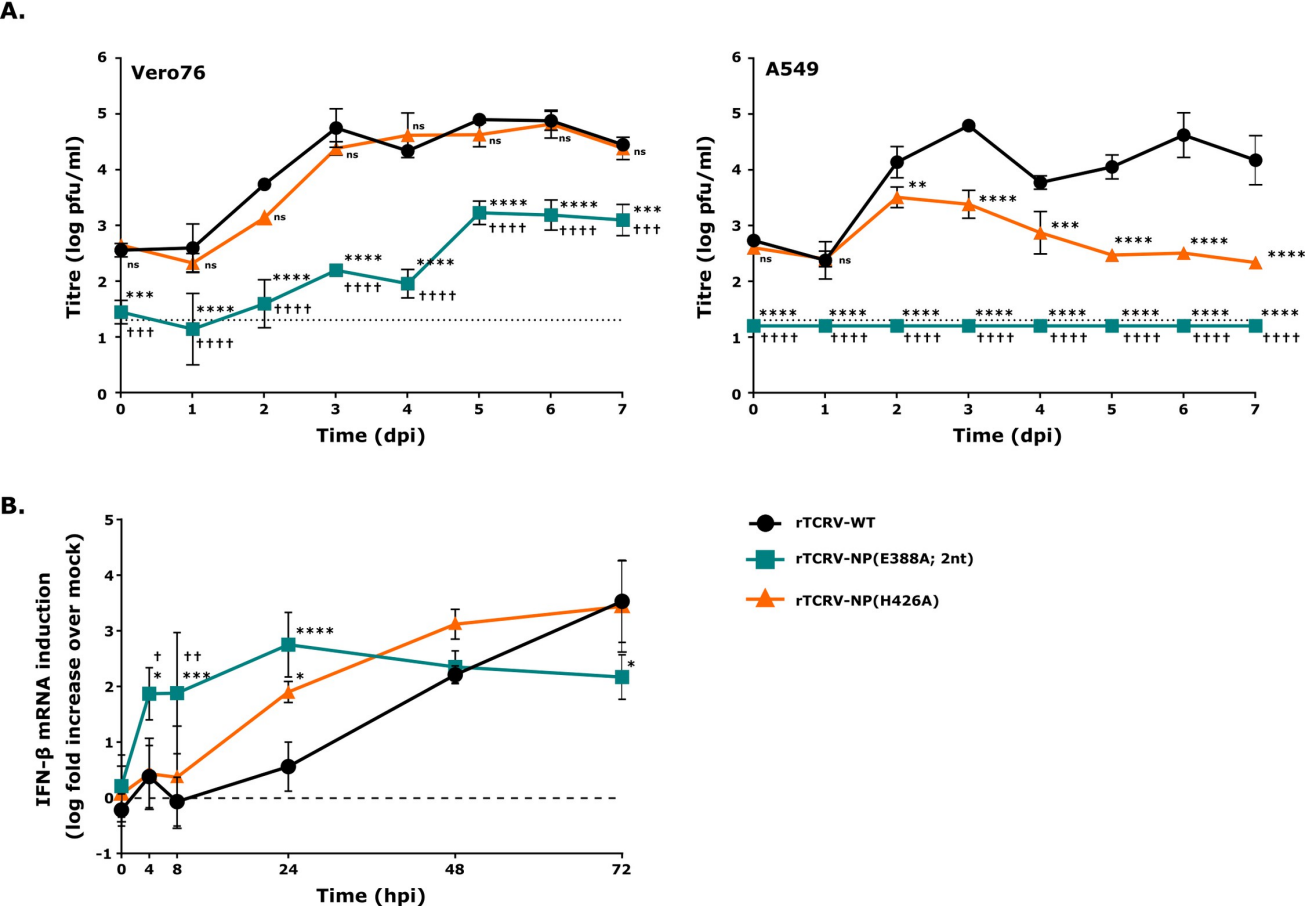
Characterization of recombinant TCRV encoding exonuclease mutants of NP. **(A) Comparison of growth kinetics.** Vero76 and A549 cells were infected at an MOI of 0.05 with either rTCRV encoding NP wild-type (rTCRV-WT) or the indicated mutants (i.e. rTCRV-NP(H426A) or rTCRV-NP(E388A; 2nt)) and supernatants were collected from day 0 to 7 for titration by plaque assay. The data are shown as means and standard deviations of 2 independent experiments. The limit of detection of the assay is indicated as a dotted line. **(B) Interferon-β mRNA induction.** A549 cells were infected as described in (A) and RT-qPCR was performed with GAPDH and IFN-β primers at the indicated time points. IFN-β mRNA levels were calculated as fold induction over mock (indicated as a dashed line) using the ΔΔCt method (i.e. standardized to GAPDH and mock). Data are shown as the means and standard deviations of three independent replicates. Statistical analysis was performed using two-way ANOVA with Dunnett’s post-hoc test for differences compared to rTCRV-WT, indicated with stars (* p≤0.05, *** p≤0.001, **** p≤0.0001, all other pairings were not significant), or using Tukey’s post-hoc test for differences between rTCRV- NP(E388A; 2nt) and rTCRV-NP(H426A), indicated with daggers († p≤0.05, †† p≤0.01, all other pairings were not significant).

Analysis of virus growth showed that the recombinant TCRV with a 2 nucleotide mutation in the active site (rTCRV-NP(E388A; 2nt)) showed markedly attenuated growth compared to a recombinant virus encoding wild-type NP (rTCRV-WT) in Vero76 cells (Fig 6A, left panel). In contrast, the recombinant TCRV with an RNA substrate-binding mutation (rTCRV-NP(H426A) grew to comparable titres and with similar kinetics to wild-type in Vero76 cells (Fig 6A, left panel). Further, consistent with their impaired ExoN activity, both mutants showed marked impairment in their growth in IFN-competent A549 cells; however, growth of the H426A mutant was still significantly more robust than for the E388A mutant, for which no detectable growth was observed at all (Fig 6A, right panel). These data support the findings of the IFN-β reporter assays, which indicated that the active site mutant (E388A) is much more significantly impaired in IFN antagonism, but also support that this mutant has additional impairments in basic aspects of its biology that affect growth independent of the effects of IFN.

Further examination of IFN-β production in A549 cells in response to infection with these viruses showed that recombinant rTCRV-WT, after producing a slight transient increase in IFN-β mRNA transcription at 4 hours post-infection (hpi), was able to successfully suppress upregulation of IFN-β transcription, which then increased only gradually during the time-frame from 24–72 hpi (Fig 6B). By comparison, the substrate RNA-binding mutant (rTCRV-NP(H426A)) showed a more rapid increase in IFN-β transcript accumulation, with levels already being markedly increased at 24 hpi (Fig 6B), at which point the levels were also significantly increased compared to WT. However, at all other time points IFN-β transcript levels were statistically similar to WT. In contrast, the active site mutant (rTCRV-NP(E388A)) already strongly upregulated IFN-β mRNA transcription at 4 hpi, and these levels remained high for the duration of the infection (i.e. up to 72 hpi) (Fig 6B). This was despite the lack of detectable virus growth in these cells (cf. Fig 6A, right panel). Not only was IFN-β transcription significantly increased in response to infection with rTCRV-NP(E388A) at 4 hpi, 8 hpi and 24 hpi compared to rTCRV-WT, but at early time points (i.e. 4 hpi and 8 hpi) it was also significantly increased compared to the substrate RNA-binding mutant rTCRV-NP(H426A). Thus, these results also appear to support a profound defect in IFN-I antagonism by the active site mutant, while the substrate RNA-binding mutant showed a much more moderate phenotype.

## Discussion

Apart from nidoviruses, arenaviruses are currently the only RNA viruses known to encode a 3′-to-5′ ExoN that degrades dsRNA. However, among nidoviruses the ExoN appears to serve primarily a proof-reading function to ensure high-fidelity replication [37,38], although recent reports now suggest that it may also have indirect consequences for resistance to IFN [39,40]. In contrast, recombinant arenaviruses, i.e. PICV and LASV, encoding mutations in the ExoN active site show clear deficits in their ability to suppress IFN-I production in response to infection [19,28]. Further, recent work has also directly shown that mutation of the ExoN active site increases dsRNA accumulation in the cytoplasm of LASV infected cells [10]. However, it has been previously observed for both PICV and LCMV that mutation of the ExoN active site not only affects the ExoN activity of NP, but also its ability to block IRF3 activation [16,19] due to effects on an overlapping DIEG(R) motif involved in IKKε-binding [15–19]. Consequently, the relative contributions of these different IFN antagonistic functions of NP have traditionally been difficult to study in isolation, and additional tools are clearly required to address the contribution of ExoN activity to IFN-I antagonism in isolation.

With respect to identifying possible strategies to develop mutants that are exclusively impaired in their ExoN activity, it was interesting to note that Tacaribe virus (TCRV) was originally reported as a possible exception to the otherwise universal observation that both pathogenic and apathogenic arenaviruses possess NP-mediated IFN antagonistic activity [18]. This was suggested to be linked to mutations, identified in early virus sequences, in a GPPT loop that both neighbors the E388A ExoN active site residue and overlaps the IKKε-binding region responsible for inhibition of IRF3 activation. Work using recombinant TCRV NP containing these mutations showed that they indeed impair IFN-I antagonism [33]. At the same time, additional sequencing work has since indicated that the isolates currently found in many laboratories as well as biorepositories (e.g. American Type Culture Collection (ATCC), Biodefense and Emerging Infections Research (BEI) Bioresources) do not contain these mutations [23,33,41,42], and thus retain both ExoN and IFN-inhibiting activity [23,33]. On the one hand this has led some researchers to dismiss these early sequences as sequencing errors [23], while others have raised the possibility that such variants may indeed exist (or have existed at some point) in specific labs, for instance as a result of extensive passaging in IFN-deficient cells [33]. Indeed, in addition to an initial 20 suckling mouse brain passages performed during the initial isolation of TCRV strain TRVL-11573 [43], additional Vero cell passages were performed to generate stocks held in early American collections [44], and then further passages in mouse brain, Vero cells (including plaque purification) and BHK-21 cells were used to generate virus stocks in the lab reporting those unusual sequences [45]. Notably, in addition to using immunologically immature mice, this passage history includes both Vero cells, which are deficient in IFN-α and β production due to deletion of the IFN-I gene cluster [46,47] and BHK21 cells, for which evidence also suggests impaired IFN production [48–52]. Consequently, such mutants could potentially offer interesting possibilities to study IFN-deficient TCRV mutants arising naturally due to adaptation. However, a forward genetics approach in which we passaged our TCRV isolate on IFN-incompetent Vero76 cells in triplicate for up to 20 passages did not result in the selection of any mutations that would be expected to affect the ExoN or other IFN-inhibition related activities of NP. Supporting these data, when we tried to directly address the theoretical feasibility of selecting a TCRV variant with mutations affecting the GPPT loop using a minigenome system we found that the GPPT>DLQL mutant was severely impaired in viral RNA synthesis (Fig 2, top panel). Further, mutation of the active site-proximal G residue that forms part of the DIEG(R) sequence involved in IKKε-binding/IRF3 inhibition [15–19] alone already severely impaired (G389A) or abolished (G389P) viral RNA synthesis. Unfortunately, efforts to restore the function of NP-DLQL in viral RNA synthesis by reverting this highly conserved G residue (i.e. GLQL), were also unsuccessful. Interestingly, unlike classical ExoN mutants affecting the active site residues (i.e. E388A), all of these mutants also showed impaired protein expression (Fig 2, bottom panel), consistent with the predicted changes in their free energy of folding (Fig 1), and in the case of the GPPT>DLQL and GPPT>GLQL mutations, also previously reported observations [33]. The effects of mutations in this region on protein expression are likely due to the position of the GPPT sequence, which forms a flexible loop between the first two strands of a β-sheet in the NP structure. Residues in both of these first two strands act to coordinate the ExoN active site cation (Mn^2+^ in the LASV structure), while the third strand coordinates a second cation (Zn^2+^ in the LASV structure) that has been suggested to play a role in structural stabilization of the C-terminal domain and/or substrate specificity [21]. Our minigenome and expression data, therefore, strongly suggested that such naturally occurring ExoN-deficient TCRVs with mutations in this active site-proximal loop would be non-viable, and consistent with this expectation we were unable to rescue any of the corresponding recombinant TCRVs. Thus, when taken together, our findings indicate that these atypical TCRV sequences that have been reported in early sequencing records indeed represent sequencing errors and are without further biological implications.

In contrast to these NP mutants focused on the GPPT active site-proximal loop, mutants of TCRV NP where the ExoN activity was destroyed by mutating one of the key active site residues (i.e. E388A) showed neither significant alterations in their expression nor their activity in viral RNA synthesis compared to wild-type (Fig 2, top panel). A similar phenotype was also observed when we examined an NP mutant targeting binding of the non-substrate strand of the ExoN dsRNA substrate (i.e. H426A). Importantly, this supports the conclusions of previous studies that ExoN activity is not directly required for viral RNA synthesis [19,21,28]. Nonetheless, some studies have suggested a role for ExoN activity in achieving optimal levels of RNA synthesis based on the observation that NP containing ExoN active site mutations show reduced activity in minigenome assays, and that the corresponding virus showed slightly impaired growth already in IFN-deficient cells [19,28]. Indeed, we also saw a very slight non-statistically significant decrease in minigenome activity for both of these NP mutants (Fig 2, top panel). However, while we saw a marked inhibition of growth in IFN-deficient cells for recombinant viruses encoding the E388A mutation, wild-type-like growth was observed for the H426A mutant (Fig 6A), suggesting that for TCRV these minor differences in activity in the minigenome assay do not reflect biologically meaningful changes in the levels of viral RNA synthesis.

The negative effects on virus growth that we observed with the active site mutant, even in IFN-independent contexts, is consistent with previous reports for other arenaviruses [19,28]; however, the effects we saw were more pronounced. One possible explanation for this observation is that, as a consequence of their impaired growth, ExoN active site mutants have been reported to show a strong tendency towards reversion both *in vitro* and *in vivo* [15,19,28]. Consistent with these reports we saw a similar tendency of our rTCRV-E388A mutant to revert after even a limited number of passages in IFN-deficient cells (S6 Fig). To overcome this issue, which otherwise greatly complicates data analysis, we used a 2-nucleotide substitution to fix the E388A mutation in our recombinant virus. The resulting lack of reversion may help to explain its somewhat more drastic phenotype compared to active site mutants used in other studies. Importantly, mutations in the ExoN active site also led to very severe growth defects in IFN-competent A549 cells. This was the case in both our own study using TCRV (Fig 6A) and in a previous report using PICV [19], with little or no growth being observed for either virus. This was in contrast to the findings using our RNA-binding mutant (H426A), which showed a much more modest growth impairment in A549 cells compared to wild-type, suggesting that this mutant retains the ability to inhibit IFN-I production in response to infection, albeit with a lower efficiency than wild-type TCRV. In this context, it is a significant advantage of the RNA-binding (H426A) mutant that we do not observe any impairment of virus growth in IFN-deficient contexts, and as such can conclude that the effects seen during growth in IFN-competent A549 cells are due solely to impairment of its ExoN activity and the resulting effects on IFN antagonism.

Excitingly, these mutants based on either classical mutation of the active site (E388A) or substrate RNA-binding (H426A) also showed dramatically different phenotypes in other assays directly examining functions related to IFN-I antagonism. As previously reported for analogous active site mutants in the background of other arenavirus NPs [16,19], the TCRV E388A mutant showed a profoundly reduced level of ExoN activity (85% loss of activity) as well as a complete loss of its ability to suppress IFN-β promoter activation in reporter assays (Figs 3 and 4). Similarly, we found that a mutation destabilizing substrate RNA-binding (i.e. H426A) also resulted in an NP that was strongly inhibited in its ExoN activity (68% loss of activity, Fig 3). This difference in the extent of ExoN impairment observed between the E388A and H426A mutants appear to be consistent with the different mechanisms underlying their functional impairment, i.e. disruption of the active site (E388A) vs. impaired protein:substrate interaction. However, despite these significant reductions in ExoN activity, the H426A mutant still efficiently suppressed IFN-β promoter activation in response to SeV infection (88% of WT inhibition levels, Fig 4).

Importantly, since the mutational approach used to generate the H426A mutant targeted substrate RNA-binding, it involves residues located away from the ExoN-proximal DIEG(R) motif associated with IKKε-binding/IRF3 inhibition (Fig 1), and as such it is reasonable to assume that it does not impact this mechanism. Indeed, biologically separable roles of ExoN activity and IKKε-binding in IFN antagonism were already hinted at by studies using chimeric PICV NP mutants containing the GPPT>DLQL mutation where, in contrast to the results with TCRV, mutation of this motif alone was not enough to abolish IFN antagonism but rather required additional mutations [33]. In the case of TCRV NP we could confirm interaction with IKKε by co-immunoprecipitation, as would be expected based on previously published data showing that these interactions are highly conserved among both NW and OW arenaviruses [16]. Interestingly, however, mutation of the ExoN active site did not seem to result in impairment of IKKε-binding, despite the mutated residue being part of the DIEG(R) motif (Fig 5). Importantly, we could show that this was not due to non-specific interaction of TCRV NP with the myc-tag used for detection of IKKε (S5 Fig). This finding is in contrast to previous work with LCMV NP, which showed that binding to IKKε was impaired by mutation of any of the ExoN active site residues [16]. Rather, our data appear to more closely resemble the situation with PICV, where mutations of the various active site resides all impaired IFN antagonism by NP, but the effects on IKKε-binding were variable [19], suggesting that for these viruses it may rather be a structural contribution of specific active site residues, rather than their role in ExoN activity, that affects IKKε interaction.

Finally, consistent with our findings using IFN-β promoter-based reporter systems, we found that infection with recombinant viruses encoding the active site mutant (i.e. E388A) resulted in a much more rapid accumulation of high levels of IFN-β mRNA than the RNA-binding mutant (i.e. H426A) (Fig 6B). This was despite the fact that, in contrast to wild-type TCRV and the RNA-binding mutant (H426A), the active site (E388A) mutant showed no detectable growth in IFN-competent A549 cells (Fig 6A). In contrast, IFN-β mRNA expression during infection with the substrate RNA-binding mutant (i.e. H426A) was only slightly elevated compared to wild-type and showed similar overall kinetics. While it must be noted that IFN-β mRNA expression does not always correlate with levels of the bioactive protein, the only regulatory mechanisms described to date by which arenavirus NP regulates IFN-β production act at the transcriptional level (reviewed in [31]). Thus, while further work remains needed to investigate the possibility of additional post-transcriptional mechanisms of IFN regulation by arenaviruses, these data strongly suggest that, in the context of TCRV infection, the ExoN activity plays only a modest role in contributing to the regulation of IFN-I production.

In addition to its scientific findings, an additional technical innovation of this work is its establishment of a non-radioactive assay for the measurement of arenavirus ExoN activity. The few studies to date that have examined ExoN activity for arenavirus NPs have made use of gel-based analysis of RNA-degradation using radioactive substrates [19,21,23,27,30]; however, such approaches have drawbacks in terms of both workload and safety. In contrast, non-radioactive assays based on the measurement of free-nucleotide release are routinely used for studies of pestivirus exonucleases [34,53–55], and offer significant advantages in both these respects. These approaches proved to be easily adaptable to studying the arenavirus NP ExoN activity and required only minor modifications of the buffer (to eliminate EDTA and accommodate an apparent preference for Mn^2+^ within the arenavirus NP ExoN active site [21]), and switching to the preferred dsRNA substrate [22,23]. Using this approach, we could clearly see ExoN activity in lysates expressing NPs from both OW and NW arenaviruses (S3 Fig) and show that these signals were reduced by treatment with a known nuclease inhibitor (ATA; S4 Fig) [35,36]. Interestingly, while studies performed in a viral context have clearly suggested that there are differences in the effectiveness with which the ExoN domains of OW and NW arenaviruses are able to degrade dsRNA [10], we did not see prominent differences when examining cell lysates transfected with different arenavirus NPs (i.e. LASV, TCRV, JUNV). However, this is actually consistent with the findings of another previous study using cell lysates expressing transfected NP [23], supporting that this assay produces results comparable to those obtained with conventional gel-based analysis of RNA-degradation. As such, we suggest that this is a suitable alternative for the analysis of arenavirus NP ExoN activity that has the potential to both increase the safety of such studies and also facilitate larger scale analyses (e.g. of larger sets of ExoN mutants) than are currently feasible. Indeed, this is supported by a proof-of-concept experiment in which we converted this assay to a high-throughput-compatible fully 96-well plate-based format. Here the data continue to support a strong separation between positive (TCRV NP(WT)) and negative (TCRV NP(E388A)) samples and produced a starting Z’-factor of 0.24 based on these initial attempts (S4 Fig). While Z’-factors >0.5 indicate excellent assays, values between 0 and 0.5 are still considered usable, and in many cases perform well in practice [56]. This suggests that while the assay could benefit from further optimization, it indeed has potential utility in high-throughput-based approaches, including drug screening. This is supported by our ability to detect dose-dependent inhibition of TCRV ExoN activity by a known inhibitor (ATA, S4 Fig) in a range of concentrations similar to those used in other studies [35,36].

Taken together, our study describes a novel approach to creating ExoN mutants of arenavirus NPs that is based on the destabilization of substrate RNA-binding (i.e. H426A) through modification of a residue located away from the active site itself. While this results in strong inhibition of ExoN activity, the ability to robustly inhibit IFN-I induction is retained both in reporter assays and during infection with recombinant TCRV. In contrast, a mutant affecting the ExoN active site directly (E388A) was profoundly impaired in both ExoN activity and its ability to inhibit IFN-β promoter activation. This difference in phenotypes is consistent with previous work showing that mutants targeting the ExoN active site also impact IKKε-mediated regulation of IRF3 activation [19,28]. Together with our own data, these findings collectively suggest that the impact of ExoN activity on NP-mediated antagonism of IFN-I responses during arenavirus infection has been significantly overestimated, and rather IRF3 inhibition may play a predominant role. Nonetheless, ExoN activity still appear to contribute to a minor extent to achieving robust IFN antagonism. Moreover, it may also play an important role in evading dsRNA detection by other RNA sensors, such as PKR, the activation of which has been suggested by several recent studies [10–12,57]. Not only do these findings significantly advance our understanding of the individual contributions these overlapping mechanisms make to IFN antagonism, but they may also have implications for therapy, and in particular suggest that recently reported approaches targeting the ExoN activity [58] may face previously unexpected challenges. Finally, looking forward, mutant viruses targeting substrate RNA-binding represent an important advancement in the available tools for performing detailed mechanistic studies into the role of ExoN activity, including with respect to IFN-I antagonism and other forms of RNA-based immune recognition, and thus will help us to better define their relevance for virus biology and pathogenesis.

## Materials and methods

### Cell culture

HEK 293T (human embryonic kidney cells, Collection of Cell Lines in Veterinary Medicine CCLV-RIE 1018), Vero76 (African green monkey kidney cells, CCLV-RIE0228) and A549 (human lung adenocarcinoma epithelial cells, CCLV-RIE1035) were cultured in Dulbecco’s modified Eagle’s medium (DMEM; ThermoFisher Scientific) supplemented with 10% fetal calf serum. BSR-T7/5 (Syrian golden hamster kidney cells, CCLV-RIE0583; kindly provided by Stefan Finke, Friedrich-Loeffler-Institut) [59] were cultured in MEM Glasgow with 10% newborn calf serum (NCS) and treated with 1 mg/ml Geneticin every other passage. Cell lines were additionally supplemented with 100 U/ml penicillin/100 μg/ml streptomycin (ThermoFisher Scientific) and 1x GlutaMAX (ThermoFisher Scientific). All cells were grown at 37°C in the presence of 5% CO2.

### Viruses

Tacaribe virus (strain TRVL-11573) was kindly provided by the University of Geneva (Dan Kolakofsky and Dominique Garcin) via the Phillips Universität Marburg (Stephan Becker). Sendai virus (SeV, strain Fushimi) grown in embryonated chicken eggs to a titer of 3x10^9^ ffu/ml was kindly provided by Stefan Finke (Friedrich-Loeffler-Institut).

### TCRV full-length clone and minigenome plasmids

The TCRV full-length plasmids were constructed using standard molecular cloning techniques based on RNA extracted from Tacaribe virus (strain TRVL-11573). Genome segments were cloned in cRNA orientation between the T7 promoter and HDV ribozyme/T7 terminator elements of the previously described vector pAmp [60]. Sequences obtained corresponded to recently published corrected TCRV (strain TRVL-11573) sequences [41] (GenBank Accession: MT081316 (S-segment) and MT081317 (L-segment)), with the exception of a unique silent genetic marker incorporated in each segment (S-segment: T2830C, ΔBamHI; L-segment: G6879A, ΔBspMI) to allow discrimination from wild-type.

To construct a corresponding TCRV minigenome, the viral open reading frames (ORFs) were removed by successive rounds of blunt-end ligation using PCR products in which the complete S segment (excepting the ORF to be deleted) had been amplified. Further, a unique pair of Type IIS cloning sites were inserted in place of each of the ORFs (BspMI for NP/L, BsmBI for GPC/Z) by adding these sequences to the ends of the primers used for deletion of the viral genes. This then allowed subsequent cloning of a NanoLuc (nLuc) reporter ORF in place of either the GPC or Z ORF, respectively (S1 Fig, panel A).

The pCAGGS-T7 and pCAGGS-NP expression plasmids have been previously published [61,62], while the pCAGGS-L expression plasmid was generated using standard cloning techniques based on RT-PCR from extracted viral RNA. Details of all cloning strategies are available upon request.

### Mutagenesis of TCRV NP

To introduce mutations into TCRV NP for protein expression, the ORF was subcloned from pCAGGS-TCRV NP into pTM1 and a C-terminal Flag-tag was added to facilitate detection. Alternatively, for virus rescue, mutations were introduced directly into pAmp-TCRV-Ssegment. The indicated mutations were introduced using site-directed mutagenesis with the iProof High Fidelity PCR kit (BioRad). After amplification, the input material was digested with DpnI prior to transformation. Primers used for mutagenesis are listed in S1 Table. As an exception, the DLQL mutation (where standard mutagenesis was unlikely to work well due to the long 12 base pair mismatch needed) was generated using conventional PCR. Here the desired changes were introduced at the ends of the primers such that the mutated sequence could be reconstituted by blunt-end self-ligation of the PCR product. This DLQL mutant served as the basis for further incorporation of the GLQL mutation, by site-directed mutagenesis, while all other mutations were introduced into the wild-type TCRV NP sequence. Following mutagenesis in pTM1, the altered NP ORFs were subcloned back into pCAGGS for expression.

### Minigenome assay

BSR-T7/5 cells were seeded in 12-well plates one day before transfection for 30–50% confluence. Transfection was performed using TransIT-LT1 (Mirus Bio) according to the manufacturer’s instructions, with complex formation performed in OptiMEM using 3 μl TransIT-LT1/μg DNA. Each sample received the following plasmids: pCAGGS-T7 (125ng), pAmp-TCRV MG-nLuc (125ng) and pCAGGS-L (500ng). In addition, samples received variable amounts of pCAGGS-NP or each mutant that resulted in a corresponding level of protein expression: pCAGGS-NP-WT (50ng), pCAGGS-NP-AGPPT (50ng), pCAGGS-NP-APPT (1000ng), pCAGGS-NP-PPPT (1000ng), pCAGGS-NP-DLQL (2000ng), pCAGGS-NP-GLQL (2000ng) or pCAGGS-NP-H426A (50ng). pCAGGS-FF (encoding Firefly (FF) luciferase, 50ng) was also added as a transfection control for normalization [61]. Additional samples were prepared without pCAGGS-NP (using empty pCAGGS to equalize the total transfected plasmid mass) to serve as a negative control. At 48 h post-transfection cells were harvested by replacing the medium with 200 μl GloLysis Buffer (Promega). They were then incubated for 5 min at room temperature (RT) before collecting the lysates and clearing them of cell debris at 12,000 x g for 5 min. Luciferase activity was measured using the NanoGlo and BrightGlo luciferase assay systems (Promega) using 40 μl of lysate and 40 μl of substrate per sample. Measurements were performed using a GloMax-Multi Microplate Reader (Promega).

### Analysis of NP expression by western blot

Cell lysates, collected as described above from minigenome assays, were mixed with 4x SDS loading buffer [200 mM Tris-HCl pH 6.8; 8% SDS; 40% Glycerol; 4% β-mercaptoethanol; 50 mM EDTA; 0.08% bromophenol blue]. Samples were separated on a 12.5% acrylamide gel before being transferred to a polyvinylidene difluoride membrane. Expression of the various NP mutants was detected either using a previously published guinea pig polyclonal anti-TCRV NP antibody raised against the C-terminal half of the protein ([63]; 1:10,000) together with a rabbit anti-guinea pig-HRP secondary antibody (Dianova, DAB-087875, 1:5,000), or mouse anti-Flag (M2) (Sigma, F1804, 1:5,000) with a secondary horse anti-mouse-HRP (Cell Signaling Technology, #7076, 1:10,000). Ebola virus (EBOV) VP35 was detected using rabbit anti-EBOV VP35 (Gentaur, 494-0301-040, 1:500) with a secondary goat anti-rabbit-HRP (Cell Signaling Technology, #7074, 1:2,000). As loading controls, actin was detected using mouse anti-actin (AC-40) (Sigma, A3853, 1:4,000) with a secondary goat-anti-mouse-HRP (Santa Cruz Biotechnology, sc-2031, 1:20,000), while GAPDH was detected using mouse anti-GAPDH (0411) (Santa Cruz, sc47724, 1:1,000) and vinculin was detected using mouse anti-vinculin (7F9) (Santa Cruz Biotechnology, sc-73614, 1:2,000), both with a secondary horse anti-mouse-HRP (Cell Signaling Technology, #7076, 1:5000). Blots were incubated with Clarity Western ECL Substrate (BioRad) and imaged by exposure to X-ray film (Fuji Super RX-N, Fujifilm).

### Exonuclease assay

HEK 293T cells in 6-well-plates with 70% confluence were transfected with 500 ng of pCAGGS encoding either Flag-tagged TCRV NP-WT, or the indicated TCRV NP mutants (except for the GPPT>DLQL mutant for which 2ug of plasmid was used) using TransIT-LT1 (Mirus Bio) according to the manufacturer’s instructions with complex formation performed in OptiMEM using 3 μl TransIT-LT1/μg DNA. After 24 h the cell culture medium was replaced and cells were allowed to incubate for a further 48h. Cells were then lysed by removing the medium and adding 150 μl of 1% Triton X-100 (Roth) after which the plates were incubated for 10 min at room temperature with intermittent shaking. Cell lysates were then centrifuged at 10,000 x g for 3 min to remove cell debris. The reaction mixture was then prepared by diluting 15 μl cell lysate and 30 μl of 10x Tris-manganese buffer [250 mM Tris-HCl, 10 mM MnCl2, 100 mM NaCl, 150 mM KCl, pH 7.66] (adapted from [23]) in DEPC-treated water (Ambion) for a total volume of 270 μl. To start the reaction, 300 μg (10μg/ul) of low molecular weight poly(I:C) (Invivogen) was added as an RNA substrate. Samples were incubated at 37°C and the reaction was stopped after 0, 4, 8, 12 and 24 h, as indicated, by mixing 50 μl of the reaction mixture 1:1 with 1.2 M perchloric acid containing 20 mM lanthanum sulfate that had been pre-chilled on ice. After a 15 min incubation on ice the samples were centrifuged for 15 min at 21,000 x g at 4°C to precipitate macromolecules (i.e. undigested substrate RNA). Supernatants were transferred into a fresh tube and the absorbance at 260nm (i.e. released nucleotides) was measured using a NanoPhotometer P300 (Implen). For each sample three repeat measurements were made to ensure precise measurements.

### Endotoxin removal from plasmids

Plasmids used in IFN-β reporter assays were cleaned with the MiraCLEAN Endotoxin Removal kit according to the manufacturer’s instructions with 3 rounds of LPS removal. Afterwards, 2 volumes of -20°C ethanol were added and the samples were incubated at -80°C for 60 min. The DNA precipitate was then pelleted by centrifugation at 14,000 x g for 20 min and washed with 500 μl of room temperature 70% ethanol. The centrifugation step was then repeated and the ethanol was removed. Plasmids were air-dried and resuspended in DEPC-treated water (Ambion).

### Interferon-β reporter assay

HEK 293T cells in 12-well plates with 70% confluence were transfected with a plasmid encoding firefly luciferase under the control of a minimal IFN-β promoter (p-125-FF, 250 ng) [64] as well as 500 ng of pCAGGS encoding Flag-tagged TCRV NP-WT, the indicated TCRV NP mutants (except for the GPPT>DLQL mutant for which 2ug of plasmid was used) or EBOV VP35. As a negative control, empty pCAGGS was transfected in the place of the viral protein-encoding plasmid. Transfection was performed using TransIT-LT1 (Mirus Bio) according to the manufacturer’s instructions with complex formation performed in OptiMEM using 3 μl TransIT-LT1/μg DNA. Additionally, pCAGGS encoding Renilla luciferase (50ng) was included as a control for transfection efficiency. Each sample was prepared in duplicate and at 24h post-transfection the cells were either infected with SeV at a multiplicity of infection (MOI) of 200 or mock infected. Cells were incubated with virus for 1 h after which DMEM + 5% FCS was added to the cells. After further incubation for 24 h, the medium was removed and 200 μl GloLysis Buffer (Promega) was added to each well to lyse the cells. Plates were then incubated for 10 min at room temperature with intermittent shaking. Cell lysates were then centrifuged at 10,000 x g for 3 min to remove cell debris. Luciferase activity was measured using the RenillaGlo and BrightGlo luciferase assay systems (Promega) using 40 μl of lysate and 40 μl of substrate per sample. Measurements were performed using a GloMax-Multi Microplate Reader (Promega).

### Coimmunoprecipitation of NP with IKKε

HEK 293T cells in 6-well plates were transfected with pCAGGS plasmids encoding IKKε-myc (1000 ng) and either Flag-tagged TCRV NP-WT or the indicated mutants (DLQL: 4000 ng, all others: 1000 ng) using TransIT LT-1 (Mirus Bio) according to the manufacturer’s instructions with complex formation performed in OptiMEM using 3 μl TransIT-LT1/μg DNA. Two days post-transfection, cells were lysed in 1 ml coIP lysis buffer [1% NP40; 50 mM Tris-HCl; 150 mM NaCl; pH7.4] with cOmplete protease inhibitor (Roche) for 2 h at 4°C. CoIPs were then performed essentially as previously described [65]. Briefly, 750 μl of the cleared lysate was subjected to immunoprecipitation using an anti-Flag antibody (mouse anti-Flag M2 (F1804), Sigma-Aldrich) and protein G-coupled Dynabeads (ThermoFisher Scientific) with 1 μl antibody per 10 μl beads. Immunoprecipitation was performed at room temperature for 10 min. Samples were washed and transferred to new tubes before heating for 10 min at 99°C. The remaining 150 μl (representing 20% of the sample used for immunoprecipitation) of the cleared pre-immune lysate was subjected to acetone precipitation. Input and coIP samples were subjected to SDS-PAGE and Western blot. For detection of TCRV NP-Flag, a mouse anti-Flag M2 (F1804, Sigma-Aldrich; 1:2000) was used together with goat anti-mouse IgG (kappa light chain)-Alexa Fluor 680 (115-625-174, Jackson Immunoresearch; 1:40.000), while for IKKε-myc, rabbit anti-myc (PA1-981, ThermoFisher; 1:1000) was used together with goat anti-rabbit IgG-Alexa Fluor 790 (111-655-144, Jackson Immunoresearch; 1:50,000). Data were analyzed using an Odyssey CLx Infrared Imaging System (LI-COR).

### Rescue of recombinant Tacaribe viruses

BSR-T7/5 cells in 6-well plates with 70–80% confluence were transfected with the following components: pAmp-TCRV Sseg-cRNA (1000 ng), pAmp-TCRV Lseg-cRNA (1000 ng), pCAGGS-T7 (250 ng), pCAGGS-L (1000 ng), pCAGGS-NP (250 ng). The transfection was performed with TransIT-LT1 (Mirus Bio) according to the manufacturer’s instructions using 5 μl TransIT-LT1/μg DNA. One day after transfection medium was exchanged against fresh MEM (Glasgow) + 2% NCS. Seven days post-transfection Vero76 cells seeded for 70–80% confluence in 6-well plates were infected with 1ml of supernatant from the transfected cells for 1h, after which DMEM + 2% FCS was added without removing the inoculum. CPE development on Vero76 cells was monitored and once significant CPE was observed, a virus stock was grown. Here a T162 flask of 70–80% confluent Vero76 cells was infected with a 1:10 dilution of virus supernatant for 1 h, after which the inoculum was removed and DMEM + 2% FCS added. The cells were incubated for 5–7 days and CPE development was monitored until approximately half of the cells were rounded up, at which point the supernatant was collected and cleared of cell debris by centrifugation for 15 min at 800 x g. An additional 5% FCS was then added and the virus was aliquoted for storage at -80°C. All rTCRV stocks were titrated by plaque assay and the full genome sequences were determined as described below.

### Virus sequencing

For sequencing of recombinant TCRVs, RNA was extracted from virus stocks using the QIAamp Viral RNA Mini kit (Qiagen) according to the manufacturer’s instructions. Full-length genome cDNA was reverse transcribed with a primer binding to the conserved 3’ genome end using the SuperScript III RT kit (Thermo Fisher), also according to the manufacturer’s instructions. Subsequent amplification of individual PCR fragments for sequencing was accomplished with the iProof High Fidelity PCR kit (BioRad) using the primers listed in S2 Table. PCR products were cleaned up using the Macherey-Nagel NucleoSpin Gel and PCR Clean-up kit after which Sanger sequencing was performed using the same primers used for amplification, as well as the additional sequencing primers indicated in S2 Table. PCR products corresponding to the TCRV genome ends were amplified from cDNA by using 3′ and 5′ rapid amplification based on ligation-anchored PCR, as previously described [41].

### Analysis of rTCRV growth kinetics

Vero76 (IFN-I-deficient) or A549 (IFN-I-competent) cells were grown in 24-well plates to 80–90% confluence. Cells were infected with rTCRV (wild-type), rTCRV-NP(E388A; 2nt) or rTCRV-NP(H426A) at an MOI of 0.05. After a 1 h incubation, the inoculum was removed and DMEM + 2% FCS was added. Cells were incubated for a further 7 days with supernatants and/or cell lysates collected at the indicated timepoints for evaluation of virus titre (by plaque assay) and IFN-β mRNA levels (by RT-qPCR), respectively.

### Plaque assay

Vero76 cells at 80–90% confluence in 12-well plates were infected with 500 μl of a 10-fold virus dilution series prepared in serum-free DMEM for 1 h, after which the inoculum was removed and the cells overlaid with a 1:1 mixture of 1.4% agarose and 2x MEM + 4% FCS. Plates were incubated for 7–10 days to allow plaques to form before addition of 0.1% crystal violet in 10% formalin. After fixing/staining overnight, excess staining solution and overlays were removed and the plaques were counted.

### IFN-β RT-qPCR assay

For analysis of IFN-β mRNA expression total RNA was extracted from infected A549 cells using the RNeasy Mini Kit (Qiagen) according to the manufacturer’s instructions. 500 ng of total RNA was then reverse transcribed with a dT_20_ primer using the SuperScript IV kit (Thermo Fisher) according to the manufacturer’s instructions. Target genes (IFN-β or GAPDH) were then amplified using 1 μl of cDNA and the previously published gene-specific primer sets for IFN-β [66] or GAPDH [67] with the PowerUp SYBR Green Master Mix kit (Applied Biosystems), also according to the manufacturer’s instructions. Reactions were performed in 96 well plates and run in an AriaMX Real-time PCR System (Agilent). Data was analyzed with the Aria 1.3 software (Agilent) using the ΔΔCt method.

### Comparison of TCRV and LASV NP ExoN active site structures

To homology model the LASV NP C-terminal domain (CTD) with co-crystallized dsRNA (PDB: 4VFU) onto the TCRV NP CTD structure (PDB: 4GVE) both structures were imported into Pymol version 2.1.0 and the “align” function was used with default settings. The alignment resulted in a root mean square deviation (RMSD) of 0.626 Å between the two crystal structures. The LASV NP CTD structure was subsequently hidden from the final view.

### Prediction of stability for TCRV NP mutants

In order to assess the stability of TCRV NP after introduction of the various mutations used in this study, FoldX v.4 [68] was used. First, the crystal structure of the TCRV NP CTD (PDB: 4GVE) was repaired using the “RepairPDB” command so that amino acids were at their local minima determined by FoldX, which resulted in a RMSD of 0.001 Å from the original structure. Next the “BuildModel” function was used to generate optimized structures of the different mutants, and calculate the ΔGibb’s free energy measure, compared to the wild-type folding energy.

### Statistical analysis

Statistical analysis was performed using GraphPad Prism 8.0.1. Data containing two parameters were compared by a two-way analysis of variance (ANOVA), while data with one variable were compared with ordinary one-way ANOVA. Post-hoc tests were selected based on the dataset: Dunnett’s (comparison to a control), Tukey’s (all possible comparisons), or Sidak’s (selected comparisons), as indicated in the respective figure legends.

## Supporting information

S1 Table Primers for mutagenesis of TCRV NP. Nucleotides that have been altered to introduce mutations into the TCRV NP sequence are shown bold-faced and underlined.
(DOCX)

S2 Table Primers for sequencing of recombinant TCRVs. (DOCX)

S1 Fig Establishment of S and L segment-based minigenome assays for TCRV. **(A) Minigenome structure.** Schematic representations of the T7-driven S segment and L segment minigenomes used in this study are shown with features to scale. Key elements include T7 promoter (T7_P_) and terminator (T7_Ter_) sequences as well as a hepatitis delta virus (HDV) ribozyme sequence. Viral elements indicated include the terminal untranslated regions (3’ UTR and 5’ UTR) and intergenic regions (IGRs). The terminal highly conserved 3’ and 5’ UTR sequences critical for viral RNA synthesis are shown boxed with nucleotides corrected according to recent updated sequencing reports (GenBank Accession #: S segment, MT081316; L segment, MT081317) highlighted in red. A cloning cassette replacing the NP or L open reading frame (ORF) with a short linker containing two BsmBI sites for cloning of foreign genes at these sites are also indicated (ΔNP and ΔL, respectively). A Nanoluciferase (NanoLuc) ORF replaces either the GPC or Z ORF in the S and L segment minigenomes, respectively. **(B) Activity of S and L segment-based minigenomes and functional evaluation of tagged NP constructs.** BSR-T7/5 cells were transfected with pCAGGS plasmids expressing TCRV NP (125ng) and TCRV L (500ng), as well as Firefly luciferase (50ng, for normalization) and T7 RNA polymerase (125ng) in addition to either an S or L segment-based TCRV minigenome (125ng), as indicated. To assess the impact of tagging TCRV NP, pCAGGS plasmids encoding N- or C-terminally Flag-tagged or an N-terminally Flag/HA-tagged version of TCRV NP were substituted for NP wild-type (WT), as indicated. Cells were lysed and luciferase activity measured 48 h post-transfection. Reporter activity in relative light units (RLU) is shown and was calculated as Nanoluciferase activity normalized to Firefly luciferase activity (left panel). Means and standard deviations are shown from two independent replicates. Statistical analysis was performed using a one-way ANOVA with Dunnett’s post-hoc test comparing samples to +L, -NP (ns–not significant, **** p≤0.0001). Cell lysates from the S segment minigenome samples were examined for expression of NP or its tagged variants by Western blot using either a polyclonal anti-TCRV NP antibody or an anti-Flag (M2) antibody, as indicated, with actin serving as a loading control (right panel). **(C) Effect of NP levels on minigenome activity.** Minigenome assays were performed, as described in (B) using an S segment-based minigenome transfected together with plasmids encoding T7 and L as well as varying amounts of pCAGGS-TCRV NP with an N-terminal Flag-tag, as indicated. Means and standard deviations of 4 independent replicates are shown (upper panel). The resulting cell lysates were also examined for NP expression by Western blot using a guinea-pig TCRV-NP polyclonal antibody (lower panel).
(TIF)

S2 Fig Quantification of nucleoprotein expression. To ensure equivalent expression between wild-type NP and its various mutants, protein lysates from samples analyzed in each of the different assays in this study, i.e. **(A) Minigenome assay** (Fig 2), **(B) Exonuclease assay** (Fig 3), **(C) Interferon reporter assay** (Fig 4) or **(D) Coimmunoprecipitation** (Fig 5) were analyzed by Western blot using a guinea-pig TCRV-NP polyclonal antibody. The resulting bands from 2–3 replicates per assay, were quantified using ImageJ and analyzed by One-way ANOVA with Dunnett’s Post-hoc test (comparison to a control) (ns—not significant, * p≤0.05).
(TIF)

S3 Fig Establishment of a non-radioactive *in vitro* exoribonuclease assay for arenavirus NP. **(A) Exonuclease activity in cell lysates containing different arenavirus NPs.** HEK 293T cells were transfected with plasmids encoding untagged wild-type NP from either TCRV, JUNV or LASV, as indicated. After 3 d cell lysates were harvested and incubated with the dsRNA substrate poly(I:C). Release of free nucleotides (A260) was then measured at the indicated time points. Background values for individual samples (at 0 h) and for a negative control sample (i.e. without NP) were subtracted from values to calculate the net A260 value for each sample. Data are shown as means and standard deviations of three independent replicates. Statistical analysis was performed using two-way ANOVA with Dunnett’s post-hoc test for differences compared to the respective 0 h samples (ns—not significant, * p≤0.05, *** p≤0.001, **** p≤0.0001). **(B) Comparison of the exonuclease activity of TCRV NP (WT) and TCRV NP (WT-Flag).** Experiments were performed as described in (A) following transfection with plasmids encoding wild-type TCRV NP with or without a Flag-tag, as indicated. Statistical analysis was performed using two-way ANOVA with Dunnett’s post-hoc test for differences compared to the respective 0 h samples (indicated with stars; * p≤0.05, ** p≤0.01) or using Sidak’s post-hoc test for differences between pairs of samples collected at the same time point (ns—not significant).
(TIF)

S4 Fig Conversion of the non-radioactive *in vitro* exoribonuclease assay to a 96-well plate format. **(A) Examination of assay reproducibility.** HEK 293T cells were transfected with plasmids encoding wild-type TCRV NP (positive control), TCRV NP(E388A) (negative control) or pCAGGS and cell lysates were harvested after 3 d, as for a standard tube-based assay. Cell lysates were then incubated with reaction buffer and the dsRNA substrate poly(I:C) as for the standard tube-based assay, but in a standard 96-well cell culture plate. After 0h (background) or 24h, 50ul of the reaction mixture was added to 50ul of 1.2 M perchloric acid containing 20 mM lanthanum sulfate that had been pre-chilled on ice in a new 96-well cell culture plate for 15 minutes. After further incubation of the samples for 2h at 4°C the plates were centrifuged for 2h at 4,000 x g at 4°C. Supernatants were transferred into a UV Clear 96-well plate (Greiner) for measurement of absorbance at 260nm (i.e. released nucleotides) using a GloMax-Multi Microplate Reader (Promega). For each sample three repeat measurements were made to ensure precise measurements. Background values (at 0 h) and values for a negative control sample (i.e. pCAGGS without NP) were subtracted to calculate the net A260 value for each sample. Data are shown as means and standard deviations of three independent replicates. The Z’-factor for the assay was calculated as previously described [56]. **(B) Inhibition of TCRV NP exonuclease activity by Aurintricarboxylic acid (ATA).** Exonuclease assays were performed as described above with cell lysates expressing TCRV NP (WT) but with the addition of ATA (10-1000uM) or an equivalent amount of DMSO, as indicated.
(TIF)

S5 Fig Co-immunoprecipitation of TCRV NP and exonuclease mutants with a control myc-tagged protein. HEK 293T cells were transfected with plasmids encoding Flag-tagged TCRV NP or its mutants, as well as a myc-tagged Ebola virus VP30, as indicated. Two days post-transfection cells were lysed and treated with RNase A. TCRV NP was then precipitated with an anti-Flag antibody and both input and precipitates were analyzed by Western blot. NP and VP30 were detected with antibodies specific for the Flag- or myc-tag, respectively. A representative result from 2 independent experiments is shown.
(TIF)

S6 Fig Reversion of recombinant TCRV encoding an NP(E388A) mutation during passaging on Vero76 cells. Recombinant TCRV was generated with an E388A mutation in NP introduced through either one (i.e. GAG→GCG; rTCRV-NP(E388A; 1nt)) or two (i.e. GAG→GCC; rTCRV-NP(E388A; 2nt)) nucleotide exchanges. Infections in Vero76 cells at an MOI of 0.05 were performed in triplicate and the relevant region was amplified by RT-PCR after each passage for sequencing. Representative sequencing chromatograms from before (p1) and after (p5) passaging are shown (top panel) with the codon corresponding to amino acid position 388 boxed. The resulting chromatograms for rTCRV-NP(E388A; 1nt) were further evaluated with respect to the relative proportion of the wild-type (A, red) and mutated (C, blue) nucleotide by integrating the area under each curve (lower panel).
(TIF)
