# Supplementary material for: A dsRNA-binding mutant reveals only a minor role of exonuclease activity in interferon antagonism by the arenavirus nucleoprotein
Source: PLoS Pathog. 2023 Jan 5;19(1):e1011049. doi: 10.1371/journal.ppat.1011049 (PMC9815661; doi:10.1371/journal.ppat.1011049)
Supplement: S1 Table — Nucleotides that have been altered to introduce mutations into the TCRV NP sequence are shown bold-faced and underlined. (DOCX) [file ppat.1011049.s001.docx]

| **Primer ID** | | **Sequence (5’-3’)** |
| --- | --- | --- |
| E388A (1nt) | fwd | ATTTGGTTGGACATCG**C**GGGACCTCCTACTGAC |
|  | rev | GTCAGTAGGAGGTCCC**G**CGATGTCCAACCAAAT |
| E388A (2nt) | fwd | ATTTGGTTGGACATCG**CC**GGACCTCCTACTGAC |
|  | rev | GTCAGTAGGAGGTCC**GG**CGATGTCCAACCAAAT |
| G389A | fwd | ATTTGGTTGGACATCGAGG**C**ACCTCCTACTGAC |
|  | rev | GTCAGTAGGAGGT**G**CCTCGATGTCCAACCAAAT |
| G389P | fwd | ATTTGGTTGGACATCGAG**CC**ACCTCCTACTGAC |
|  | rev | GTCAGTAGGAGGT**GG**CTCGATGTCCAACCAAAT |
| GPPT>DLQL | fwd | **CCAACT**TGACCCAGTAGAGCTTGCATTATATCAACC |
|  | rev | **AGGT**CCTCGATGTCCAACCAAATAGTGTTGG |
| GPPT>GLQL | fwd | GGTTGGACATCGAGG**G**CCTCCAACTTGACCC |
|  | rev | GGGTCAAGTTGGAGG**C**CCTCGATGTCCAACC |
| H426A | fwd | GGGATTTAAAAATGGGAGCAGA**GC**TTCTCACGGCATTCTAATGAAG |
|  | rev | CTTCATTAGAATGCCGTGAGAA**GC**TCTGCTCCCATTTTTAAATCCC |
