# Supplementary material for: A dsRNA-binding mutant reveals only a minor role of exonuclease activity in interferon antagonism by the arenavirus nucleoprotein
Source: PLoS Pathog. 2023 Jan 5;19(1):e1011049. doi: 10.1371/journal.ppat.1011049 (PMC9815661; doi:10.1371/journal.ppat.1011049)
Supplement: S2 Table — (DOCX) [file ppat.1011049.s002.docx]

|  | **Target** | **Primer** | **Sequence (5’-3’)** |
| --- | --- | --- | --- |
| PCR Product Amplification | S-segment | TCRV S 3’ end fwd | CGCACAGTGGATCCTAGGC |
|  |  | TCRV S 1906 rev | CTATTTGGCATCGTAGACATTAGG |
|  |  | TCRV S 1680 fwd | CCTGATGAACCTCACTGTGCACTG |
|  |  | TCRV S 5’end rev | CGCACCGGGGATCCTAGG |
|  | L-segment | TCRV L 3’end fwd | GGATCCTAGGCGGCACTTGAC |
|  |  | TCRV L 2292 rev | CAACATCATTCCTTCTTGAGC |
|  |  | TCRV L 1728 fwd | GATGCTACTCAATTTACAC |
|  |  | TCRV L 4664 rev | GGCACGCATAATATCTTGC |
|  |  | TCRV L 4487 fwd | GGTTATCGATTGCAGAGG |
|  |  | TCRV L 5’end rev | GGATCCTAGGCGTTACGTGC |
| Additional Sequencing Primers | S-segment | TCRV S 455 fwd | GGTACCAATGCTTACATGG |
|  |  | TCRV S 639 rev | CTACATTGTTCATAGTCTCC |
|  |  | TCRV S 1087 fwd | GCGTAGATCTGACCAAGAAGC |
|  |  | TCRV S 1695 fwd | TGCACTGCTGGACTGCATAATG |
|  |  | TCRV S 2257 fwd | CTTGGAAGAGAGTGCTGTCC |
|  |  | TCRV S 2685 fwd | TGTGTTTGTGTGGTCAGCC |
|  |  | TCRV S 2884 rev | GATGGGACCAGGACCCAGG |
|  |  | TCRV S 2993 fwd | CCAAGTTTAGGAGAGTGAACAAAGACATC |
|  | L-segment | TCRV L 633 fwd | GATACACAGTCTTTCAAACG |
|  |  | TCRV L 834 rev | CAGAAGCTCTGATCTATTAG |
|  |  | TCRV L 1183 fwd | CACTGGACTCTTTGAGAAGG |
|  |  | TCRV L 1358 rev | GATAAATCAGGATATCTTGC |
|  |  | TCRV L 2157 fwd | CTCTTAAATGTAAGTTACC |
|  |  | TCRV L 3455 rev | CAGCTTAGTGTTAAGATCC |
|  |  | TCRV L 3902 fwd | GGAGAGATACCAAGTCAC |
|  |  | TCRV L 4064 rev | ATCATCTGAAGCACAAGGC |
|  |  | TCRV L 5175 fwd | GAGCCACAGATTCCAGTGG |
|  |  | TCRV L 5311 rev | GCAAATCGCTCATGAAGG |
|  |  | TCRV L 5746 fwd | GATGTTTATTGAAGACATAGC |
|  |  | TCRV L 5912 rev | GATGTTCTAGAATGCTCC |
|  |  | TCRV L 6359 fwd | TACAGAGTGGAGTTGACTGG |
|  |  | TCRV L 6456 rev | CTTGTTGGAATAGCATCAC |
