# Supplementary figures and images for: A dsRNA-binding mutant reveals only a minor role of exonuclease activity in interferon antagonism by the arenavirus nucleoprotein
Source: PLoS Pathog. 2023 Jan 5;19(1):e1011049. doi: 10.1371/journal.ppat.1011049 (PMC9815661; doi:10.1371/journal.ppat.1011049)

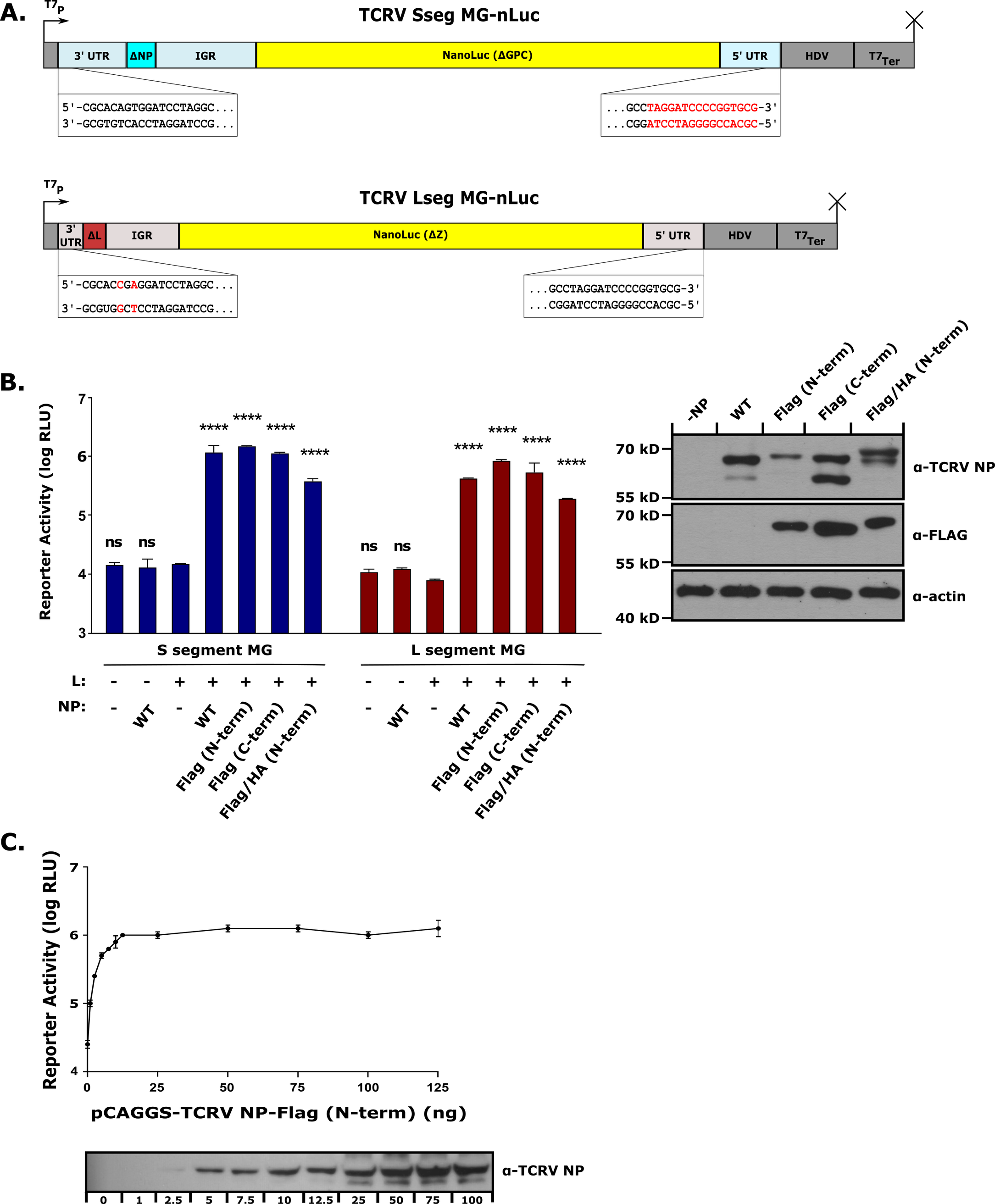

Supplement: S1 Fig — (A) Minigenome structure. Schematic representations of the T7-driven S segment and L segment minigenomes used in this study are shown with features to scale. Key elements include T7 promoter (T7P) and terminator (T7Ter) sequences as well as a hepatitis delta virus (HDV) ribozyme sequence. Viral elements indicated include the terminal untranslated regions (3’ UTR and 5’ UTR) and intergenic regions (IGRs). The terminal highly conserved 3’ and 5’ UTR sequences critical for viral RNA synthesis are shown boxed with nucleotides corrected according to recent updated sequencing reports (GenBank Accession #: S segment, MT081316; L segment, MT081317) highlighted in red. A cloning cassette replacing the NP or L open reading frame (ORF) with a short linker containing two BsmBI sites for cloning of foreign genes at these sites are also indicated (ΔNP and ΔL, respectively). A Nanoluciferase (NanoLuc) ORF replaces either the GPC or Z ORF in the S and L segment minigenomes, respectively. (B) Activity of S and L segment-based minigenomes and functional evaluation of tagged NP constructs. BSR-T7/5 cells were transfected with pCAGGS plasmids expressing TCRV NP (125ng) and TCRV L (500ng), as well as Firefly luciferase (50ng, for normalization) and T7 RNA polymerase (125ng) in addition to either an S or L segment-based TCRV minigenome (125ng), as indicated. To assess the impact of tagging TCRV NP, pCAGGS plasmids encoding N- or C-terminally Flag-tagged or an N-terminally Flag/HA-tagged version of TCRV NP were substituted for NP wild-type (WT), as indicated. Cells were lysed and luciferase activity measured 48 h post-transfection. Reporter activity in relative light units (RLU) is shown and was calculated as Nanoluciferase activity normalized to Firefly luciferase activity (left panel). Means and standard deviations are shown from two independent replicates. Statistical analysis was performed using a one-way ANOVA with Dunnett’s post-hoc test comparing samples to +L, -NP (ns–not signific [file ppat.1011049.s003.tif]

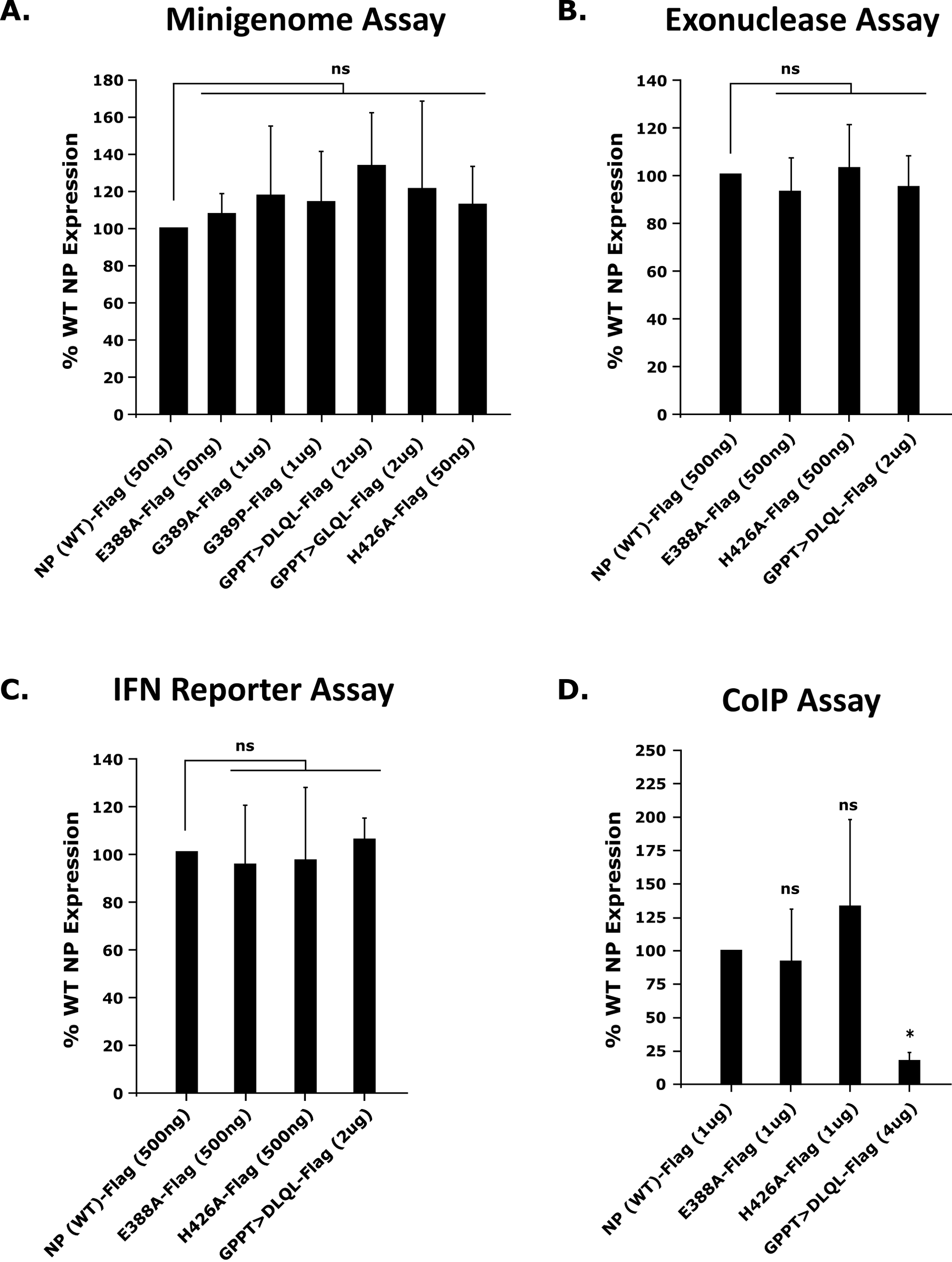

Supplement: S2 Fig — To ensure equivalent expression between wild-type NP and its various mutants, protein lysates from samples analyzed in each of the different assays in this study, i.e. (A) Minigenome assay (Fig 2), (B) Exonuclease assay (Fig 3), (C) Interferon reporter assay (Fig 4) or (D) Coimmunoprecipitation (Fig 5) were analyzed by Western blot using a guinea-pig TCRV-NP polyclonal antibody. The resulting bands from 2–3 replicates per assay, were quantified using ImageJ and analyzed by One-way ANOVA with Dunnett’s Post-hoc test (comparison to a control) (ns—not significant, * p≤0.05). (TIF) [file ppat.1011049.s004.tif]

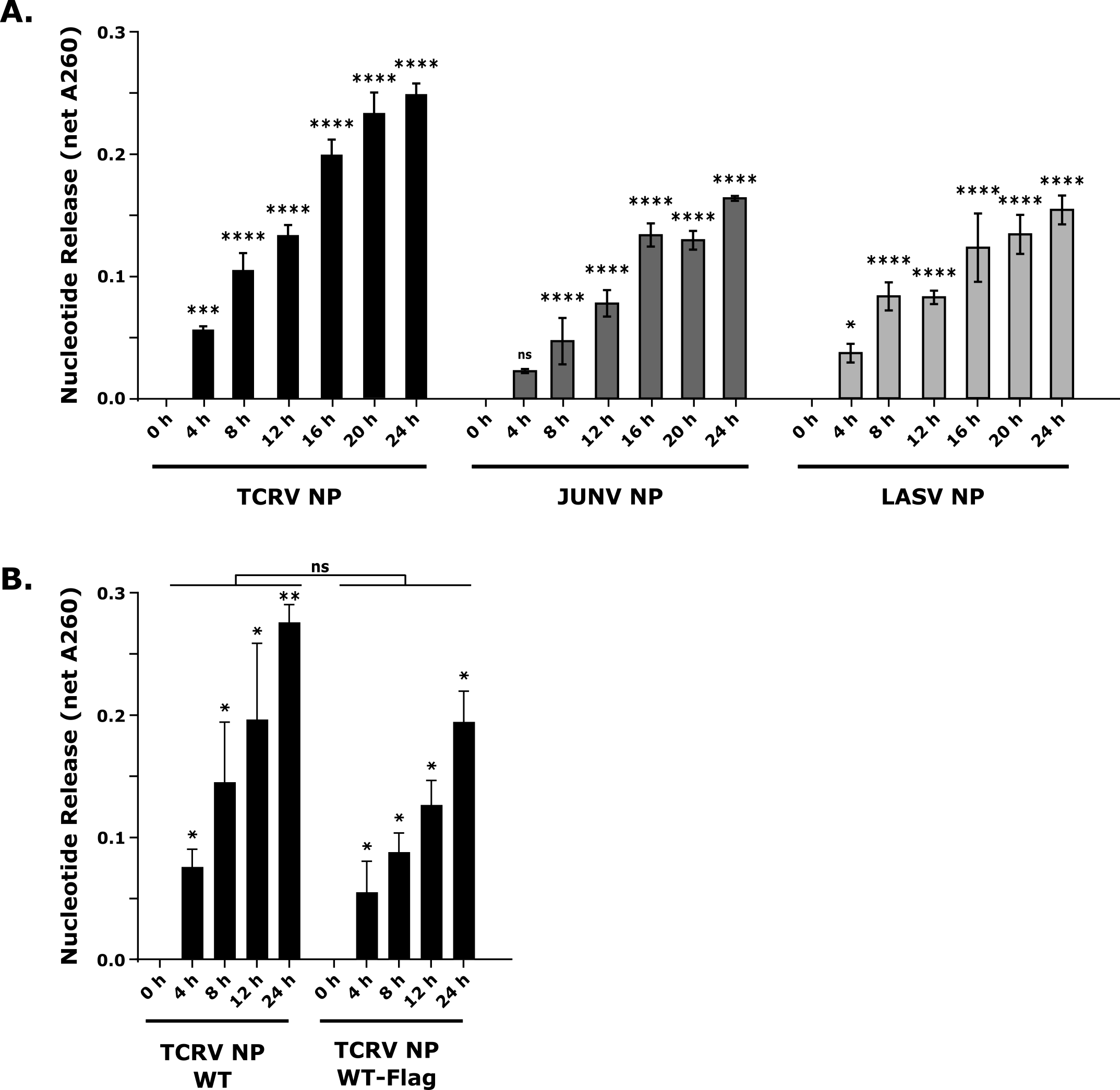

Supplement: S3 Fig — (A) Exonuclease activity in cell lysates containing different arenavirus NPs. HEK 293T cells were transfected with plasmids encoding untagged wild-type NP from either TCRV, JUNV or LASV, as indicated. After 3 d cell lysates were harvested and incubated with the dsRNA substrate poly(I:C). Release of free nucleotides (A260) was then measured at the indicated time points. Background values for individual samples (at 0 h) and for a negative control sample (i.e. without NP) were subtracted from values to calculate the net A260 value for each sample. Data are shown as means and standard deviations of three independent replicates. Statistical analysis was performed using two-way ANOVA with Dunnett’s post-hoc test for differences compared to the respective 0 h samples (ns—not significant, * p≤0.05, *** p≤0.001, **** p≤0.0001). (B) Comparison of the exonuclease activity of TCRV NP (WT) and TCRV NP (WT-Flag). Experiments were performed as described in (A) following transfection with plasmids encoding wild-type TCRV NP with or without a Flag-tag, as indicated. Statistical analysis was performed using two-way ANOVA with Dunnett’s post-hoc test for differences compared to the respective 0 h samples (indicated with stars; * p≤0.05, ** p≤0.01) or using Sidak’s post-hoc test for differences between pairs of samples collected at the same time point (ns—not significant). (TIF) [file ppat.1011049.s005.tif]

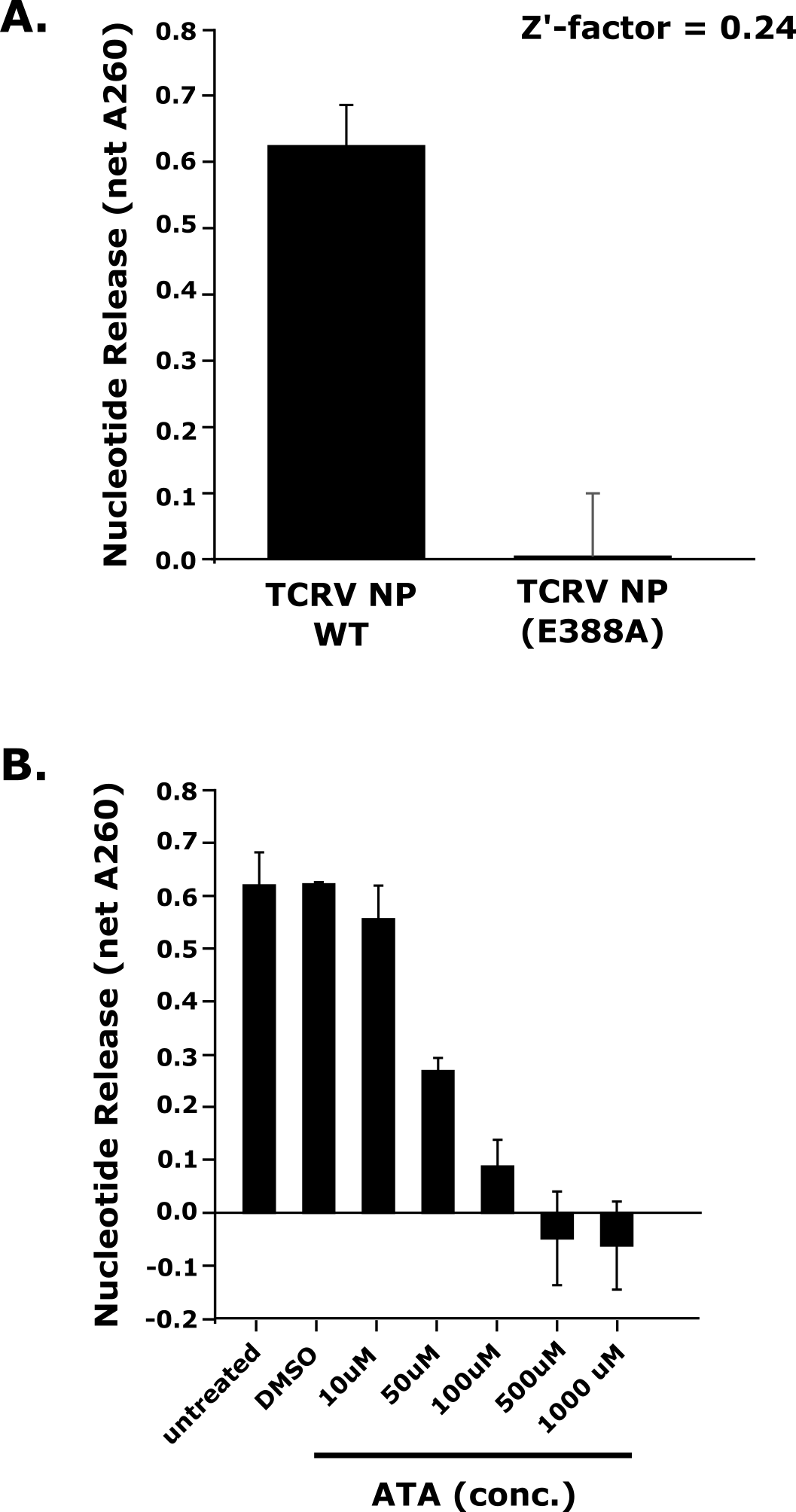

Supplement: S4 Fig — (A) Examination of assay reproducibility. HEK 293T cells were transfected with plasmids encoding wild-type TCRV NP (positive control), TCRV NP(E388A) (negative control) or pCAGGS and cell lysates were harvested after 3 d, as for a standard tube-based assay. Cell lysates were then incubated with reaction buffer and the dsRNA substrate poly(I:C) as for the standard tube-based assay, but in a standard 96-well cell culture plate. After 0h (background) or 24h, 50ul of the reaction mixture was added to 50ul of 1.2 M perchloric acid containing 20 mM lanthanum sulfate that had been pre-chilled on ice in a new 96-well cell culture plate for 15 minutes. After further incubation of the samples for 2h at 4°C the plates were centrifuged for 2h at 4,000 x g at 4°C. Supernatants were transferred into a UV Clear 96-well plate (Greiner) for measurement of absorbance at 260nm (i.e. released nucleotides) using a GloMax-Multi Microplate Reader (Promega). For each sample three repeat measurements were made to ensure precise measurements. Background values (at 0 h) and values for a negative control sample (i.e. pCAGGS without NP) were subtracted to calculate the net A260 value for each sample. Data are shown as means and standard deviations of three independent replicates. The Z’-factor for the assay was calculated as previously described [56]. (B) Inhibition of TCRV NP exonuclease activity by Aurintricarboxylic acid (ATA). Exonuclease assays were performed as described above with cell lysates expressing TCRV NP (WT) but with the addition of ATA (10-1000uM) or an equivalent amount of DMSO, as indicated. (TIF) [file ppat.1011049.s006.tif]

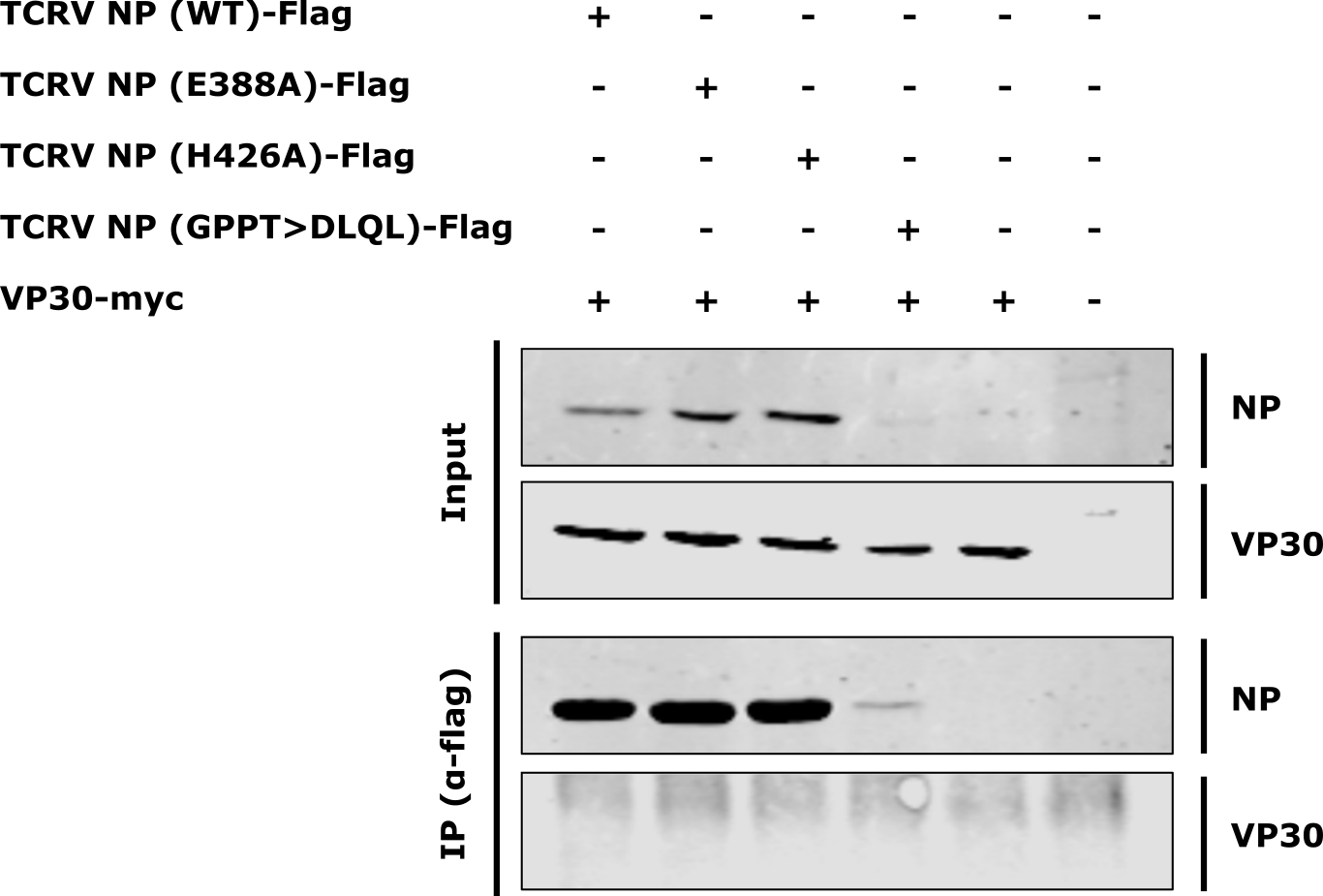

Supplement: S5 Fig — HEK 293T cells were transfected with plasmids encoding Flag-tagged TCRV NP or its mutants, as well as a myc-tagged Ebola virus VP30, as indicated. Two days post-transfection cells were lysed and treated with RNase A. TCRV NP was then precipitated with an anti-Flag antibody and both input and precipitates were analyzed by Western blot. NP and VP30 were detected with antibodies specific for the Flag- or myc-tag, respectively. A representative result from 2 independent experiments is shown. (TIF) [file ppat.1011049.s007.tif]

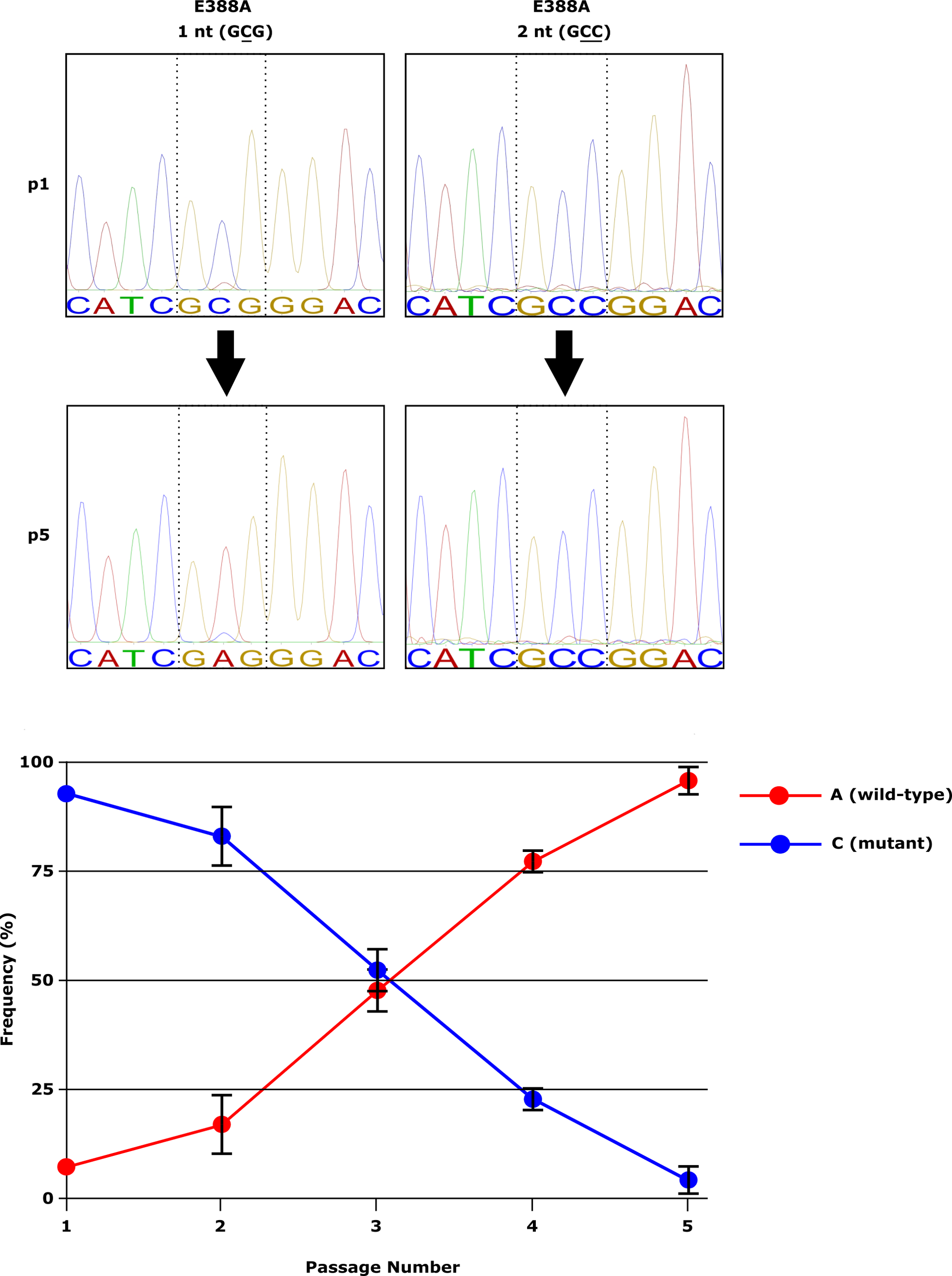

Supplement: S6 Fig — Recombinant TCRV was generated with an E388A mutation in NP introduced through either one (i.e. GAG→GCG; rTCRV-NP(E388A; 1nt)) or two (i.e. GAG→GCC; rTCRV-NP(E388A; 2nt)) nucleotide exchanges. Infections in Vero76 cells at an MOI of 0.05 were performed in triplicate and the relevant region was amplified by RT-PCR after each passage for sequencing. Representative sequencing chromatograms from before (p1) and after (p5) passaging are shown (top panel) with the codon corresponding to amino acid position 388 boxed. The resulting chromatograms for rTCRV-NP(E388A; 1nt) were further evaluated with respect to the relative proportion of the wild-type (A, red) and mutated (C, blue) nucleotide by integrating the area under each curve (lower panel). (TIF) [file ppat.1011049.s008.tif]
